# Clinical characteristics of 27 children with febrile infection‐related epilepsy syndrome in a single center

**DOI:** 10.1002/pdi3.84

**Published:** 2024-06-09

**Authors:** Juan Wang, Yongfang Liu, Lingling Xie, Min Cheng, Lianying Feng, Yuhang Liu, Yi Guo, Li Jiang

**Affiliations:** ^1^ Department of Neurology Children's Hospital of Chongqing Medical University National Clinical Research Center for Child Health and Disorders Ministry of Education Key Laboratory of Child Development and Disorders Chongqing Key Laboratory of Child Health and Nutrition Chongqing China; ^2^ Chongqing Key Laboratory of Pediatrics Chongqing Key Laboratory of Child Health and Nutrition Chongqing China; ^3^ Department of Nutrition Children's Hospital of Chongqing Medical University Chongqing China

**Keywords:** children, FIRES, ketogenic diet, trajectory analysis

## Abstract

To investigate the clinical characteristics of febrile infection‐related epilepsy syndrome (FIRES). We used trajectory analysis and logistic regression analysis to investigate the clinical characteristics and prognostic risk factors respectively. Twenty‐seven patients (16 males) were included. The median age of onset was 7 (IQR: 4–9) years. Routine cerebrospinal fluid (CSF) examination was normal. Electroencephalogram (EEG) showed frequent microseizures and electroseizures in all patients. Eight patients had claustrum signs in the acute phase. Anesthetics and anti‐seizure medications (ASM) were used in all patients. All patients received immunotherapy, including plasma exchange (*n* = 4), immunoglobulin (*n* = 26), and corticosteroids (*n* = 19). Trajectory diagrams of seizure showed 6 patients had bimodal disease course. Besides, we found there may be a linear relationship between body temperature and convulsion frequency (*R* ^2^ = 0.25). The median Glasgow outcome scale (GOS) was 3 (IQR: 1–4). Nine deaths occurred, including abandonment of treatment (*n* = 3), hemodynamic instability (*n* = 3), brain hernia (*n* = 2), and brain hernia with hemodynamic instability (*n* = 1). Seizure onset combined with fever (*p* = 0.003), periodic discharge (*p* = 0.002), and non‐ketogenic diet (non‐KD) (*p* = 0.005) were independent risk factors for death. The KD group (*n* = 10) had lower mortality (*p* = 0.009), lower convulsion frequency at latest follow‐up (*p < *0.001), less ASM (*p* = 0.002), and higher GOS (*p < *0.001) than non‐KD group (*n* = 17). Therefore, some FIRES patients may have bimodal disease course. There may be a linear relationship between body temperature and convulsion frequency. Seizure onset combined with fever, periodic discharge and KD may affect the prognosis.

## Graphical abstract

Our febrile infection‐related epilepsy syndrome (FIRES) patients had a poor prognosis with a mortality up to 33%. Seizure onset combined with fever, periodic discharges and ketogenic diet (KD) may affect the prognoses of FIRES patients. Among the above 3 independent risk factors for death, KD is a controllable factor for clinicians.

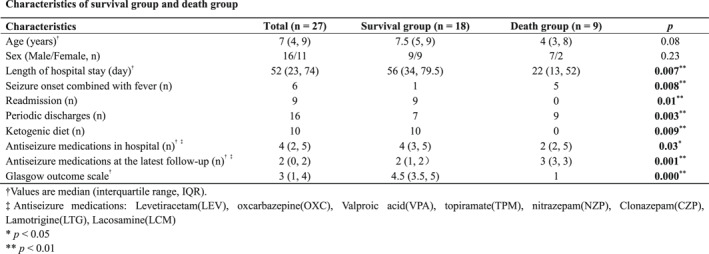

## 1 INTRODUCTION

Febrile infection‐related epilepsy syndrome (FIRES) is a subset of new onset refractory status epilepticus (NORSE). In acute phase (1–12 weeks), the seizure burden and mortality are high, and in chronic phase drug‐resistant multifocal epilepsy and intellectual disability exist.^1^ FIRES are rare and severe, and have a high mortality rate. Currently, clinical management of FIRES is difficult due to lack of specific biomarkers, widespread drug resistance, and poor knowledge. Nonpharmacological therapy such as ketogenic diet (KD) is effective for some FIRES, and the International Expert Consensus on FIRES published in 2022 points the way for the diagnosis and treatment of FIRES,^2^ including the initiation of first‐line immunotherapy such as plasma exchange (PE), immunoglobulin (IG), corticosteroids (CS) treatment within 3 days, starting second‐line treatment such as KD within 1 week. Nevertheless, the efficacy of IL‐1R blockers and IL‐6 blockers need to be further confirmed. Studies have shown that fever may affect the prognosis of super‐refractory status epilepticus (SRSE),^3^ but the factors affecting the prognosis of FIRES are not very clear. At present, FIRES studies were mostly reported as case report.^4^, ^5^, ^6^, ^7^ The largest sample size reported by international multi‐centers was 77 in 2011.^8^ This study obtained the efficacy of KD and found that burst suppression of EEG was associated with cognitive impairment. However, no risk factors for death were identified. In view of the rarity of FIRES, many doctors fail to recognize the disease. In some hospitals, the reporting time of metabolic and genetic test is long, and it is difficult to initiate KD within 1 week. Therefore, there may be a gap between the real world and the latest expert consensus. In this study, real‐world trajectory analysis was applied to study the clinical characteristics of FIRES, and logistic regression analysis was used to explore the factors affecting prognosis.

## 2 MATERIALS AND METHODS

### 2.1 Experimental design

We retrospectively collected 27 patients diagnosed with FIRES hospitalized in the Children's Hospital Affiliated to Chongqing Medical University from 2015 to 2022 and analyzed their clinical characteristics and related factors. The diagnostic criteria met the international expert consensus in 2022.^2^ This study was approved by the Ethics Committee of the Affiliated Children's Hospital of Chongqing Medical University.

### 2.2 Clinical data collection

The collected data included the following: (1) general information: age, sex, onset time, course of disease at referral, time to peak seizure frequency, duration of status epilepticus (SE), length of hospital stay, and diagnosis time; (2) the relationship between fever and seizure: the time from fever onset to the first seizure, seizure onset combined with fever, maximum daily body temperature and seizure frequency; (3) electroencephalogram (EEG) characteristics: ictal phase, interictal discharge and background; (4) imaging findings: including cerebral computed tomography (CT) or magnetic resonance imaging (MRI) findings; (5) cerebrospinal fluid (CSF) test results: CSF cell count, protein, sugar, chloride, antibody and cytokines; (6) treatment: immunotherapy, KD, ventilator, sedatives, anesthetics, muscle relaxants, and vasoactive drugs; and (7) prognosis: seizure control, anti‐seizure medications (ASM), ventilation and reventilation were needed, whether the patient died, whether the patient was readmitted, and the Glasgow Outcome Scale (GOS) prognosis score at the latest follow‐up.

### 2.3 Implementation of the KD

After contraindications to a KD were excluded, the classic KD regimen was applied to patients. The KD was initiated through a gradual, nonfasting transition based on the recommendations of the International Ketogenic Diet Study Group.^9^ A commercial formula (Jiantong, GB/T29602) for tube or oral feeding was advised at the beginning of the diet to achieve stable blood ketone levels, followed by a switch to a more flexible diet of home‐prepared food. The KD ratio started at 2:1 and was adjusted depending on individual tolerance, seizure reduction and serum beta‐hydroxybutyric acid (BHB) levels.

According to experience and consensus on KD from the China Association Against Epilepsy,^10^ the treatment goal was ≥50% seizure reduction accompanied by a serum BHB level of 1.2–4.9 mmol/L. When adequate seizure reduction was not achieved at low BHB levels, the KD ratio was increased to achieve a BHB level of 4–5 mmol/L.

### 2.4 Statistical analysis

In this section, (1) SPSS18.0 software was used for statistical analysis. Continuous variables were expressed as median (interquartile range, IQR). Categorical variables were expressed as count (percent). (2) Linear regression analysis was used to analyze the linear relationship between fever and seizure frequency. The Pearson test and Spearman test were used to test the correlation. (3) The Pearson test was used to analyze the correlation between prognosis and clinical characteristics. The survival group and the death group, the KD group and the non‐KD group were compared. The *t* test was used to compare normally distributed measurement data. The Mann‐Whitney *U* test was used to compare nonnormally distributed measurement data, and Fisher's exact analysis was used to compare count data. (4) Multivariate logistic regression analysis was used to analyze the risk factors for death. The Hosmer‐Lemeshow test was used to determine the goodness of fit. If *p* < 0.05, it was considered to be a significant difference.

## 3 RESULTS

### 3.1 General information

**FIGURE 1:**
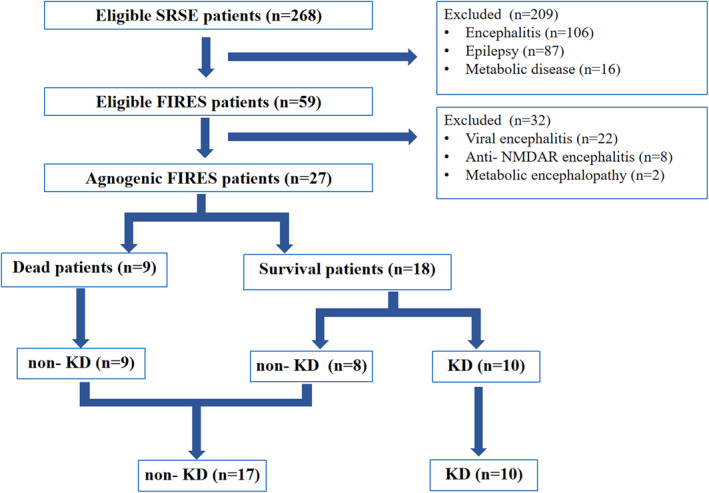
Flowchart of data screening and grouping. We screened 27 FIRES from 59 eligible FIRES among 268 SRSE patients, including 10 patients with KD and 17 patients without KD. FIRES, febrile infection‐related epilepsy syndrome; KD, ketogenic diet; SRSE, super‐refractory status epilepticus.

A total of 27 patients with FIRES had no positive previous history. Data screening and grouping are detailed in Figure 1. There were 16 males and 11 females. The median age was 7 (IQR: 4–9) years. The median time to referral was 4 (IQR: 2–8) days. The median length of hospital stay was 52 (IQR: 23–74) days. Ten patients were treated with KD, and 17 were treated with non‐KD. Eventually, 18 patients survived, and 9 died. The death group (*n* = 9) and the survival group (*n* = 18), and the KD group (*n* = 10) and the non‐KD group (*n* = 17) were compared. In order of the onset time of convulsions, survivors are numbered S‐1 to S‐18, deaths are numbered D‐1 to D‐9, and details of trajectory analysis for each patient are shown in Supporting Information S1.

**FIGURE 2:**
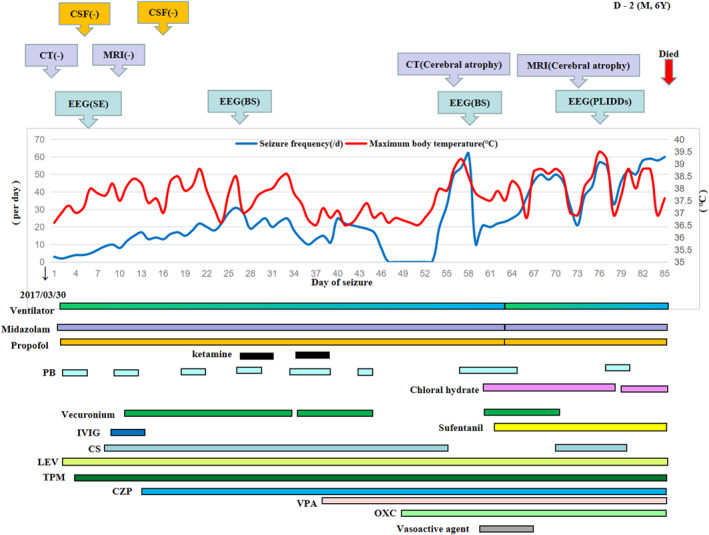
Trajectory diagram of patient D‐2. Patient D‐2 is male, 6 years old. He had seizure onset on 30 March 2017, and he died on 85th day of the disease course. From this figure, we concluded there may be a linear relationship between the highest daily body temperature (red line) and the convulsion frequency (blue line). Additionally, he appeared bimodal course on the 53th day of the disease. In terms of the convulsion frequency, the second peak was higher than the first peak. Two tests of cerebrospinal fluid (CSF) were normal. Cerebral computed tomography (CT) and magnetic resonance imaging (MRI) were normal in the beginning and became cerebral atrophy in the end. Electroencephalogram (EEG) showed burst suppression (BS) and periodic long‐interval diffuse discharges (PLIDDs). CS, Corticosteroids; CZP, Clonazepam; IVIG, Immunoglobulin; LEV, Levetiracetam; OXC, Oxcarbazepine; PB, Phenobarbital; TPM, Topiramate; VPA, Valproic acid.

From the trajectory chart (see Supporting Information S1), we found 6 patients (S‐2, S‐5, S‐18, D‐2, D‐5, D‐7) had a bimodal course of “onset‐remission‐relapse”, accompanied by fever relapse. Four patients (S‐2, D‐2, D‐5, D‐7) had a higher convulsion frequency in the second peak compared with that in the first peak, and only one patient (S‐2) survived. Two patients (S‐5, S‐18) had a lower convulsion frequency in the second peak than that in the first peak, and they survived. For example, bimodal appeared in patient D‐2 on the 53rd day of the disease course, the convulsion frequency of the second peak was higher than that of the first peak, and he died on the 85th day of the disease course (see Figure 2).

### 3.2 Relationship between fever and convulsions

**FIGURE 3:**
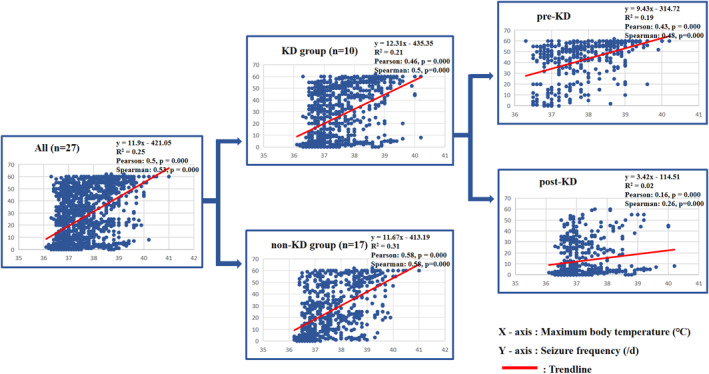
Linear chart between body temperature and convulsion frequency. We found a linear relationship between the highest daily body temperature and the convulsion frequency (*R* ^2^ = 0.25, Pearson coefficient = 0.50, Spearman coefficient = 0.53, *p < *0.001). The linear relationship was significant in both KD group (*R* ^2^ = 0.21, Pearson coefficient = 0.46, Spearman coefficient = 0.5, *p < *0.001) and non‐KD group (*R* ^2^ = 0.31, Pearson coefficient = 0.58, Spearman coefficient = 0.58, *p < *0.001). To analyze the effect of KD, the data of the KD group was divided into pre‐KD and post‐KD. As can be seen in Figure 3, the linear relationship of pre‐KD was significant (*R* ^2^ = 0.19, Pearson coefficient = 0.43, Spearman coefficient = 0.48, *p < *0.001), but the linear relationship of post‐KD was nonsignificant (*R* ^2^ = 0.02, Pearson coefficient = 0.157, Spearman coefficient = 0.26, *p < *0.001). Besides, the slope of trend line (intercept) was decreased during post‐KD than pre‐KD (3.42 vs. 9.43).

**FIGURE 4:**
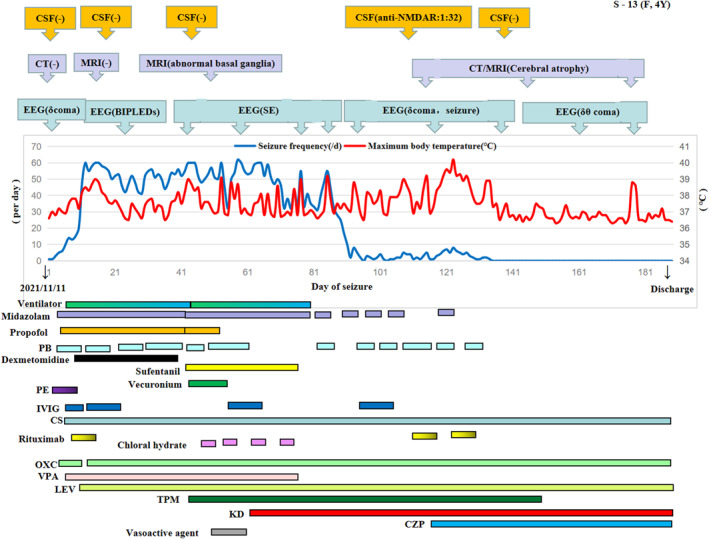
Trajectory diagram of patient S‐13. Patient S‐13 was female and 4 years old. She had seizure onset on 11 November 2021, and she developed anti‐NMDAR encephalitis at 116th day of the FIRES course (cerebrospinal fluid anti‐NMDAR antibody 1:32). In this chart, the horizontal axis represents the course of the disease (days), the left vertical axis represents the frequency of convulsions, and the right vertical axis represents body temperature. From this figure, we can see the slope of the line decreased after KD. Even with recurrent fever during anti‐NMDAR encephalitis, but his convulsions did not relapse. Besides, both brain CT and MRI were normal at the acute phase and became cerebral atrophy at the chronic phase. Electroencephalogram (EEG) showed bilateral independent periodic lateralized epileptiform discharges (BIPLEDs). CS, corticosteroids; CZP, Clonazepam; IVIG, Immunoglobulin; KD, Ketogenic diet; LEV, Levetiracetam; OXC, Oxcarbazepine; PB, Phenobarbital; PE, Plasma exchange; TPM, Topiramate; VPA, Valproic acid.

**FIGURE 5:**
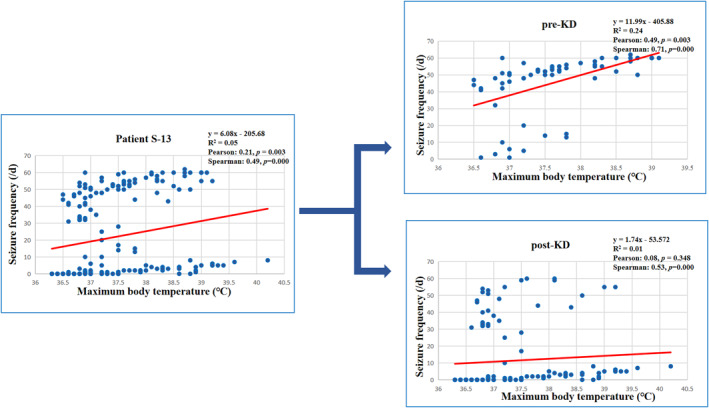
Line chart of patient S‐13. There was a linear relationship between the body temperature and the convulsion frequency of this child before KD (*R* ^2^ = 0.24), and the slope of the trend line and the statistical significance decreased after KD (11.99 vs.1.74, *R* ^2^ = 0.01).

The median interval between fever and convulsions of 27 children was 5 (IQR: 4–8) days. Six patients had seizure onset combined with fever. All patients had focal seizures with unconsciousness, generalized tonic‐clonic seizures, and status convulsions. The highest median clinical frequency of convulsions was 60 (57, 60) times per day. By statistical analysis, we found a linear relationship between the highest daily body temperature and the convulsion frequency (*R* ^2^ = 0.25; Pearson coefficient = 0.50, Spearman coefficient = 0.53, *p < *0.001). The linear relationship was significant in both KD group (*R* ^2^ = 0.21; Pearson coefficient = 0.46, Spearman coefficient = 0.50, *p < *0.001) and non‐KD group (*R* ^2^ = 0.31; Pearson coefficient = 0.58, Spearman coefficient = 0.58, *p < *0.001). To analyze the effect of KD, the data of KD group was divided into pre‐KD and post‐KD. As can be seen in Figure 3, the linear relationship of pre‐KD was significant (*R* ^2^ = 0.19; Pearson coefficient = 0.43, Spearman coefficient = 0.48, *p < *0.001), but the linear relationship of post‐KD was nonsignificant (*R* ^2^ = 0.02; Pearson coefficient = 0.157, Spearman coefficient = 0.26, *p < *0.001). Besides, the slope of trend line (intercept) was decreased during post‐KD than pre‐KD (3.42 vs. 9.43). We have also investigated the linear relationship for each patient. For example, as can be seen in Figure 4, patient S‐13 developed anti‐NMDAR encephalitis on the 116th day of the FIRES course (cerebrospinal fluid anti‐NMDAR antibody 1:32). During anti‐NMDAR encephalitis, even if there was recurrent fever, the frequency of convulsions did not relapse. There was a linear relationship between body temperature and convulsion frequency in this child before KD (*R* ^2^ = 0.24), and the slope of trend line decreased after KD (11.99 vs. 1.74). After KD, the linear relationship was not significant (*R* ^2^ = 0.01) (see Figure 5). The details of each person can be seen in Supporting Information S2.

### 3.3 Electroencephalogram (EEG)

All 27 patients underwent 4‐h video electroencephalogram (VEEG) examination. All EEGs were abnormalities as *δ*/*θ* coma, multifocal discharge, focal seizures, and SE. The median clinical seizure frequency was 5 (3, 9) per 4 h, but the median EEG seizure frequency was 33 (22, 44) per 4 h, because there were microseizures and undetected electrical seizures in EGG, the number of episodes captured by EEG was greater than the clinically recorded (*p < *0.001). Besides, we found 16 patients had periodic discharges (PD), 11 had burst suppression (BS), 5 had bilateral independent periodic lateralized epileptiform discharges (BIPLEDs), 4 had periodic long‐interval diffuse discharges (PLIDDs), and 4 had BS with BIPLEDs/PLIDDs.

### 3.4 Imaging

All 27 patients underwent cerebral CT/MRI. At the onset of disease, 27/27 (100%) images were negative. In the acute phase, 10/27 patients (37%, S‐1, S‐7, S‐8, S‐10, S‐11, S‐14, S‐17, D‐1, D‐3, D‐9) were considered negative, and 17 patients were abnormal as follows: 6 patients (S‐3, S‐5, S‐9, S‐18, D‐2, D‐7) showed cerebral atrophy, 6 patients (S‐13, S‐16, D‐4, D‐5, D‐6, D‐8) showed abnormal basal ganglia, 4 patients (S‐11, S‐12, S‐15, D‐4) showed abnormal bilateral temporal lobe, 3 patients (S‐2, S‐4, D‐7) showed abnormal bilateral cerebral lobe, 2 patients (D‐6, D‐8) showed edema, and one patient (D‐6) showed a cerebral hernia. We retrospectively reviewed the MRI images, and found 8/27 (30%) patients (S‐4, S‐6, S‐7, S‐12, S‐13, S‐16, D‐4, D‐6) had Claustrum signs in the acute phase, and 8 patients had abnormalities in T 1WI, T 2WI, and FLAIR. Six patients (S‐4, S‐6, S‐12, S‐16, D‐4, D‐6) had abnormal DWI. Two patients (S‐7, S‐13) had normal DWI. The above abnormal signs disappeared in the chronic phase. The 18 survivors had MRI images in the chronic phase, 13 abnormal and 5 negative at discharge. At the latest follow‐up, 16 patients showed brain atrophy and 2 patients (11%) were negative. In addition, patient S‐11 was found normal on CT but abnormal on MRI in the same phase, please see the 11th figure in Supporting Information S1 for details.

### 3.5 Cerebrospinal fluid (CSF)

In 27 patients, CSF cell count, biochemistry, etiology, and antibodies were normal in the acute phase. Two of them (S‐13, S‐14) had abnormal CSF immunotests: (1) On the 116th day of the disease course, patient S‐13 showed repeated body temperature and increased involuntary movement, and reexamination of anti‐NMDAR antibody in CSF suggested 1:32. Her CSF returned to normal at 138th day in the disease course. (2) Patient S‐14 showed increased CSF IL‐8 (127.8 pg/mL; reference range: 0–47 pg/mL) and MCP‐1 (548.7 pg/mL; reference range: 70–127 pg/mL) at 11th day in the disease course. The CSF test was repeated after 2 weeks of KD, then IL‐8 level decreased to 50.7 pg/mL, which parallels with her improvement in convulsion control and physical condition, while MCP‐1 level increased to 703.7 pg/mL. Her serum cytokines were normal before KD and remained normal at 1 and 2 weeks after KD.

### 3.6 Treatment

#### 3.6.1 Immunotherapy

All 27 patients were treated with PE, IVIG, and CS either alone or together. Three patients (S‐1, S‐5, S‐13) were treated with PE (Once a day for 3 times for patient S‐1; once every other day for 3 times for patient S‐5 and patient S‐13). Except for patient S‐1, 26 patients had at least one time IVIG application, with a total of 2 g/kg in 2–5 days. Five patients (S‐4, S‐6, S‐9, S‐18, D‐5) received IVIG 2 times, 2 patients (S‐2, S‐13) received IVIG 3 times, and the intervals were more than 3 weeks. Nineteen patients (S‐2, S‐4, S‐5, S‐6, S‐8, S‐11, S‐12, S‐13, S‐14, S‐15, S‐16, S‐17, S‐18, D‐2, D‐5, D‐6, D‐7, D‐8, D‐9) received methylprednisolone 10–20 mg/k/d for 3–5 days. Then, they received prednisone orally at 1.5–2 mg/kg/d. This dose was maintained for 1 month and then gradually reduced to discontinuation. The median course was 54 (IQR: 30–80) days. One patient (S‐13) was treated with rituximab 3 times on the 7th, 116th, 123th day in the disease course at 375 mg/m^2^ per time.

#### 3.6.2 KD treatment

Ten patients (S‐2, S‐4, S‐5, S‐6, S‐9, S‐10, S‐11, S‐12, S‐13, S‐14) were treated with KD. The median disease course of KD initiation was 36 (IQR: 19–60) days, and the KD ratios of 4:1, 3:1, and 2:1 were 5, 2, and 3 respectively. One patient (S‐11) was initially given intravenous ketogenesis, then switched to gastric tube feeding and finally to oral feeding. The remaining 9 patients were initially fed through a gastric tube and later switched to oral feeding. The median ketone level was 2.0 (IQR: 1.4–2.8) mmol/L and the median glucose level was 3.9 (IQR: 3.7–4.5) mmol/L, and the KD onset time was 6 (IQR: 4–10) days. The latest follow‐up was 22 (IQR: 18–55) months, the median KD course was 11 (IQR: 3–16) months and 6 patients had stopped KD. No patients experienced hypoglycemia (blood glucose <2.2 mmol/L) or high levels of ketosis with KD. Patients defecate 1–4 times a day. Vitamin D and blood calcium levels were normal. Patient S‐1 had mild hyperlipidemia with 2.72 mmol/L (reference 0.1–2.59 mmol/L), high‐density lipoprotein 0.54 mmol/L (reference >1.68 mmol/L), total cholesterol 3.67 mmol/L (reference <5.2 mmol/L) and triglyceride 1.25 mmol/L (reference <1.69 mmol/L). Patient S‐3 developed vomiting with KD. These abnormalities improved spontaneously without special treatment, but renal color ultrasound and electrocardiogram were both normal. There was no case of diarrhea, constipation, or abdominal distension requiring medical intervention.

#### 3.6.3 Drugs

All 27 patients had midazolam pumping starting on day 1 (IQR: 1–2), the total course was 30 (IQR: 15–39) days, and the rate was 5–30 μg/kg/min. Nineteen patients had propofol pumping starting on day 1 (IQR: 1–11), the total course was 15 (IQR: 6–30) days, and the rate was 3–5 mg/kg/h. Twenty patients had sufentanil pumping starting on day 1 (IQR: 1‐1), the total course was 30 (IQR: 16–32) days, and the rate was 0.05–0.12 μg/kg/h. Twenty‐one patients received vecuronium starting on day 6 (IQR: 2–11), the total course was 6 (IQR: 5–7) days, and the rate was 1‐2 μg/kg/min. Nine patients (S‐1, S‐9‐S‐10, S‐13, S‐16, D‐2, D‐4, D‐5, D‐6) received vasoactive drugs starting on day 10 (IQR: 5–31), and the total course was 6 (IQR: 3–10) days. Two patients (D‐4, D‐5) used vasoactive drugs for 2 periods: (1) patient D‐4 used vasoactive drugs for 1 day starting on day 4 of the disease course and for 2 days starting on day 10. (2) Patient D‐5 used vasoactive drugs for 8 days starting on day 7 of the disease course and for 2 days starting on day 28. During hospitalization, all 27 patients underwent ASM, including levetiracetam (LEV), oxcarbazepine (OXC), valproic acid (VPA), topiramate (TPM), nitrazepam (NZP), clonazepam (CZP), lamotrigine (LTG), and lacosamine (LCM). The median number of antiepileptic drugs was 4 (IQR: 2–5). Twenty‐three patients received LEV at a maintenance dose of 40 (IQR: 35–50) mg/kg/d. Twenty‐two patients received OXC at a maintenance dose of 30 (IQR: 30–38) mg/kg/d, and a drug concentration of 12 (IQR: 10–16) ug/mL. Twenty‐two patients received VPA at a maintenance dose of 30 (IQR: 28–35) mg/kg/d, and a drug concentration of 82 (IQR: 76–92) ug/mL. Eight patients received TPM at a maintenance dose of 4 (IQR: 3–5) mg/kg/d. Eight patients received NZP at a maintenance dose of 0.5 (IQR: 0.4–0.5) mg/kg/d. Five patients received CZP at a maintenance dose of 0.2 (IQR: 0.2–0.3) mg/kg/d. One patient received LTG at a maintenance dose of 5 mg/kg/d, and a drug concentration of 14.3 ug/mL. One patient received LCM at a maintenance dose of 8 mg/kg/d. All patients were treated with dehydrating agents including mannitol, glycerol fructose and hypertonic saline.

#### 3.6.4 Mechanical ventilation

Except for 2 patients (S‐12, S‐17), 25 patients received mechanical ventilation treatments with a median ventilator time of 19 (IQR: 11–31) days. Three patients (S‐15, D‐5, D‐7) received secondary ventilation treatment. Among them, 2 patients (D‐5, D‐7) died from secondary ventilation due to a bimodal course, and the two ventilation intervals were 22 and 8 days respectively. One patient (S‐15) was briefly removed from the ventilator for 2 days and was again mechanically ventilated at the beginning of the acute phase, and this patient survived.

### 3.7 Prognosis

The follow‐up period was 37 (IQR: 25–62) months. All 27 patients had median GOS score of 3 (IQR: 1–4). Four patients (S‐16, D‐1, D‐6, D‐8) developed brain herniation. Two patients (S‐16, D‐5) developed drug rashes. Among 9 deaths, three were hemodynamic instability (D‐2, D‐4, D‐5), three were abandoned treatment (D‐3, D‐7, D‐9), two were cerebral herniation (D‐1, D‐8), and one patient (D‐6) had herniation with hemodynamic instability. Two patients (D‐4, D‐5) died after two periods of vasoactive drug treatment. Those who abandoned the treatment died 1–3 h later. Among the latest follow‐up of 18 survivors showed that 8 were seizure free, 2 had a convulsion frequency of 1 per month, 3 had 2 to 3 seizures per week, and 5 had 1 to 8 seizures per day. The median ASM was 2 (0, 2). Nine patients (S‐3, S‐5, S‐7, S‐8, S‐9, S‐9, S‐13, S‐15, S‐16) were readmitted for the reonset of epilepsy (*n* = 8) and pneumonia (*n* = 1). At the latest follow‐up, 4 children (S‐5, S‐9, S‐10, S‐15) with MRI cerebral atrophy recovered normalized cognition and returned to school.

#### 3.7.1 Death factors

The correlation between death and clinical characteristics was assessed by Pearson's test. Death was negatively associated with KD (*p* = 0.003), length of stay (*p* = 0.03), the peak time of convulsions (the interval from the first convulsion to the maximum convulsion frequency) (*p* = 0.041), seizure onset combined with fever (*p* = 0.002), and periodic discharge (*p* = 0.001).

Comparing the death group (*n* = 9) and survival group (*n* = 18), there was no significant difference in age, sex, duration of convulsion, ventilator time, sedatives, anesthetics, vasoactive drugs, or immunotherapy (*p* > 0.05). The death was less than the surviving with KD (0 vs. 10, *p* = 0.009), had more seizure onset combined with fever (5 vs. 1, *p* = 0.008), had more periodic discharges (9 vs. 7, *p* = 0.003), had a shorter length of stay (22 vs. 562 days, *p* = 0.007), had fewer ASM applications (2 vs. 4, *p* = 0.03), and had a worse GOS prognosis score (1 vs. 4.5, *p* < 0.001) (see Table 1).

**TABLE 1:** Characteristics of survival group and death group.

Characteristics	Total (*n* = 27)	Survival group (*n* = 18)	Death group (*n* = 9)	*p*
Age (years)^a^	7 (4, 9)	7.5 (5, 9)	4 (3, 8)	0.08
Sex (Male/Female, *n*)	16/11	9/9	7/2	0.23
Interval from fever onset to the first seizure (day)^a^	5 (4, 8)	5 (2.75, 7.25)	6 (4.5, 11.5)	0.21
Course of disease at referral (day)^a^	4 (2, 8)	5 (2.75, 8)	4 (2, 24.5)	0.68
Time to peak seizure frequency (day)^a^	7 (2, 9)	6 (2, 8.25)	8 (2, 27)	0.41
Duration of status epilepticus (day)^a^	30 (16, 52)	34 (21.5, 48.25)	22 (13, 52)	0.25
Length of hospital stay (day)^a^	52 (23, 74)	56 (34, 79.5)	22 (13, 52)	0.007**
Time of diagnosis (day)^a^	20 (14, 35)	21.5 (15.75, 35.25)	18 (13, 38.5)	0.38
Time of ventilation (day)^a^	19 (11, 31)	20 (10, 33.5)	19 (12.5, 29)	0.98
Ketogenic diet (*n*)	10	10	0	0.009**
Seizure onset combined with fever (*n*)	6	1	5	0.008**
Bimodal course (*n*)	6	3	3	0.37
Second peak worse than first peak (*n*)	4	1	3	0.09
Reventilation (*n*)	3	1	2	0.25
Cerebral herniation (*n*)	4	1	3	0.09
Drug rash (*n*)	2	1	1	1
Readmission (*n*)	9	9	0	0.01**
Propofol (*n*)	19	11	8	0.2
Sufentanil (*n*)	21	12	9	0.07
Vecuronium (*n*)	21	13	8	0.63
Vasoactive drugs (*n*)	9	5	4	0.42
Plasma exchange (*n*)	4	3	1	1
Immunoglobulin (*n*)	26	17	9	1
Corticosteroids (*n*)	19	13	6	1
Abnormal images (*n*)	12	7	5	0.45
Periodic discharges (*n*)	16	7	9	0.003**
Anti‐seizure medications in hospital (*n*)^a^ ^,^ ^b^	4 (2, 5)	4 (3, 5)	2 (2, 5)	0.03*
Anti‐seizure medications at the latest follow‐up (*n*)^a^ ^,^ ^b^	2 (0, 2)	2 (1, 2)	3 (3, 3)	0.001**
Glasgow outcome scale^a^	3 (1, 4)	4.5 (3.5, 5)	1	0.000**

^a^ Values are median (interquartile range, IQR).

^b^ Anti‐seizure medications: Levetiracetam (LEV), oxcarbazepine (OXC), Valproic acid (VPA), topiramate (TPM), nitrazepam (NZP), Clonazepam (CZP), Lamotrigine (LTG), and Lacosamine (LCM).

*p < 0.05 **p < 0.01.

To evaluate the mortality risk factors utilizing the multivariate logistic regression analysis, seizure onset combined with fever (*p* = 0.003), periodic discharge (*p* = 0.002), and non‐KD (*p* = 0.005) were considered independent risk factors for mortality. Besides, the Hosmer‐Lemeshow test for goodness of fit suggests good (*p* = 1).

#### 3.7.2 Efficacy of KD

Comparing the KD group (*n* = 10) and non‐KD group (*n* = 17), there was no difference in sex, duration of convulsion, ventilator time, sedatives, anesthetics, vasoactive drugs, immunotherapy, or ASM application in the acute phase (*p* > 0.05). The KD group was older than the non‐KD group (8.5 vs. 5 years, *p* = 0.034), long length of stay (66.5 vs. 34 days, *p* = 0.013), and had fewer deaths (0 vs. 9, *p* = 0.009). At the latest follow‐up, 8 patients in the KD group were seizure free, and 0 patient in the non‐KD group was seizure free. There was a lower seizure frequency in the KD group than in the non‐KD group (0 vs. 3 times per day, *p < *0.001), fewer ASM (2 vs. 3, *p* = 0.002), and a high GOS score (5 vs. 1, *p < *0.001) (see Table 2). Nine patients were readmitted. Among them, 3 (S‐5, S‐9, S‐13) were in the KD group and 6 (S‐3, S‐7, S‐8, S‐15, S‐16, S‐18) were in the non‐KD group. In the KD group, one patient (S‐5) was readmitted for recurrence of convulsion at the chronic phase, and she used a KD for only 10 days at the acute phase. One patient (S‐9) had recurrent convulsion for carbohydrate consumption. One patient (S‐13) was readmitted due to pneumonia with fever but no convulsions. Unlike patients in the KD group, 6 patients in the non‐KD group were readmitted for afebrile convulsion recurrence.

**TABLE 2:** Characteristics of KD group and non‐ KD group.

Characteristics	Total (*n* = 27)	KD group (*n* = 10)	Non‐ KD group (*n* = 17)	*p*
Age (years)^a^	7 (4, 9)	8.5 (6.75, 9)	5 (4, 7.5)	0.034*
Sex (Male/Female, *n*)	16/11	6/4	10/7	1
Interval from fever onset to the first seizure (day)^a^	5 (4, 8)	6.5 (3.5, 8.25)	5 (4, 7)	0.667
Course of disease at referral (day)^a^	4 (2, 8)	4.5 (2, 17)	4 (2, 9.5)	0.899
Time to peak seizure frequency (day)^a^	7 (2, 9)	5.5 (2, 9.25)	7 (2, 13.5)	0.86
Duration of status epilepticus (day)^a^	30 (16, 52)	43 (24.5, 84.25)	23 (13.5, 47.5)	0.056
Length of hospital stay (day)^a^	52 (23, 74)	66.5 (46.75, 159.75)	34 (18, 55)	0.013*
Time of diagnosis (day)^a^	20 (14, 35)	31 (16, 52.25)	18 (13.5, 29)	0.067
Time of ventilation (day)^a^	19 (11, 31)	27.5 (14.5, 49.25)	16 (10.5, 27)	0.167
Death (n)	9	0	9	0.009**
Seizure onset combined with fever (*n*)	6	0	6	0.057
Bimodal course (*n*)	6	2	4	1
Second peak worse than first peak (*n*)	4	1	3	1
Reventilation (*n*)	3	0	3	0.274
Cerebral herniation (*n*)	4	0	4	0.264
Drug rash (*n*)	2	0	2	0.516
Readmission (*n*)	9	3	6	1
Propofol (*n*)	19	8	11	0.666
Sufentanil (n)	21	8	13	1
Vecuronium (*n*)	21	8	13	1
Vasoactive drugs (*n*)	9	3	6	1
Plasma exchange (*n*)	4	2	2	0.613
Immunoglobulin (*n*)	26	10	16	1
Corticosteroids (*n*)	19	8	11	0.666
Abnormal images (*n*)	12	5	7	0.706
Periodic discharges (*n*)	16	4	12	0.224
Anti‐seizure medications in hospital (*n*)^a^ ^,^ ^b^	4 (2, 5)	4 (3, 5)	3 (2, 5)	0.64
Anti‐seizure medications at the latest follow‐up (*n*)^a^ ^,^ ^b^	2 (0, 2)	2 (0, 2)	3 (3, 3)	0.002**
Glasgow outcome scale^a^	3 (1, 4)	5 (4, 5)	1 (1, 2)	0.000**

^a^ Values are median (interquartile range, IQR).

^b^ Anti‐seizure medications: Levetiracetam (LEV), oxcarbazepine (OXC), Valproic acid (VPA), topiramate (TPM), nitrazepam (NZP), Clonazepam (CZP), Lamotrigine (LTG), Lacosamine (LCM).

*p < 0.05 **p < 0.01.

## 4 DISCUSSION

We found there was a slight male predominance (16 males vs. 9 females), which was also reported by International League Against Epilepsy (ILAE).^1^ Our FIRES patients had a poor prognosis with a mortality up to 33%, higher than the 10% mortality in expert consensus,^2^ and comparable to the 30% reported by Dome Nico Serino.^8^ Additionally, it has been reported that the mortality rate of cerebral hernia in the state of SE was as high as 5/5 (100%).^11^ For our patients, 3/4 (75%) died from cerebral hernia. Our patient had hemodynamic instability in 4/9 (44%) deaths, which was higher than the 28% mortality rate associated with hemodynamic instability due to heart disease alone.^12^ Two of them (D‐4, D‐5) died in the second segment of vasoactive drug. One patient (D‐5) had a drug eruption, which limited the application of drugs and KD, finally died. Three thirds (100%) of our patients who abandoned the treatment died within 1–3 h, confirming that the natural course of FIRES is not optimistic. Previous work reported the high frequency of generalized tonic‒clonic seizures, age ≤14 years old, and long duration of epilepsy are independent risk factors for mortality.^13^ Nevertheless, all the above factors affected the patients in our study.

We found that PD was an independent risk factor for death of our patients having PD as much as 59% and BS up to 41%. The incidence of PD was much higher than the incidence of 25% in ICU patients reported previously.^14^ Peluso's study also suggested that EEG background is associated with neurological prognosis.^15^ For epilepsy, the appearance of PD suggested poor prognosis,^16^ and PD was significantly associated with death.^17^, ^18^ Hence, we speculate that the high mortality rate in this study may be related to the high incidence of PD in our patients. As we all know, PD can occur in many critically ill patients and is not a characteristic of the EEG of FIRES. Besides, anesthetics can induce some PD such as BS.^19^, ^20^ In our study, the incidence of BS was similar to previous studies.^21^ That study also suggested that BS patients have less convulsions than non‐BS patients, and anesthetic‐induced BS does not increase the risk of death. In contrast, our patients had uncontrolled convulsions after BS, which is related to the extensive resistance of FIRES including resistance to anesthetics. We had 4 patients with BS with BIPLEDs/PLIDDs, which may be the mixed results of the disease itself and anesthesia. We speculate that the PD of the disease itself may indicate a poor prognosis. However, it is difficult to judge whether the disease itself or anesthesia produces PD.

We found that seizure onset combined with fever was an independent risk factor for death, which is consistent with previous reports.^3^ At present, the etiology of FIRES and whether fever occurs in the first convulsion are both not clear. We also found the non‐KD treatment is a risk factor because seizure onset with or without fever may affect the diagnosis and the treatment of the disease. In general, for convulsions with fever, infectious diseases should be paid more attention, and KD should be less recommended. While for afebrile convulsions, epilepsy and KD are more suggested to consider.

A KD has been found to be effective in treating FIRES.^22^ Again, we demonstrated the good efficacy of KD for FIRES. In our study, KD not only controlled convulsions in FIRES but also significantly reduced the application of ASM and improved the GOS score. The course of disease at the initiation of KD in our patient was 16–91 days, suggesting that the initiation of KD was effective even at the course of disease up to 2–3 months. Patients with late introduced KD (S‐2, S‐4) recovered not as well as other patients with earlier KD treatment. This phenomenon suggests that the early introduction of KD may be beneficial to improve prognosis. Previous findings also support the early introduction of KD.^23^ At present, many physicians lack awareness of FIRES, and KD is severely underused in most hospitals in underdeveloped areas. Therefore, it is necessary to raise doctors' awareness of the disease and to increase the use of KD earlier in primary hospitals to benefit more patients.

Low BHB levels may cause the underestimated benefits of KD. The consensus on KD from the China Association Against Epilepsy regarding the optimal state of ketosis suggests that urine ketone level should be maintained above 3, blood ketone level should be 1.2–4.9 mmol/L, blood glucose level should be controlled at approximately 4.0 mmol/L, and blood glucose/ketone ratio (glucose/ketone index) should be 1:1–2:1.^10^ Because the definition of ketosis varies, some studies maintained a goal of a serum BHB level of 4–6 mmol/L in refractory epilepticus (RE) patients,^24^ some maintained a goal of >2–3 mmol/L in SRSE patients,^25^, ^26^ and some maintained a goal of ≥1.5 mmol/L in FIRES/SRSE patients.^27^ From these studies, we can see that the BHB value in FIRES patients was lower than that in RE and SRSE patients. In this study, the serum BHB level in the FIRES patients was 2.0 (IQR: 1.4–2.8) mmol/L. However, the treatment of the KD was good. Our results suggest that there may be an inexact correspondence between serum BHB levels and KD benefits. Some studies have shown that the classical KD results in higher blood ketone level than a modified Atkins diet.^24^ We found that two patients (S‐3, S‐8) had a lower level of ketosis (BHB 1–2 mmol/L, urine ketone 2+) even with the classical 4:1 KD but their SE stopped, and they regained consciousness and could be taken off the ventilator, which was similar to the result of the study in which two SRSE patients got benefits at lower levels of ketosis (neither urine ketone ≥2+ nor serum B O H ≥ 2 mmol/L).^26^ This finding may suggest that low level of ketosis may still reach the threshold for seizure control for a few FIRES patients. Since there is no consensus on the threshold of ketosis, it may vary among individuals.

In this study, two patients failed to adhere to the KD regimen and six patients in the non‐KD group were readmitted to the hospital due to recurrent seizures. Meanwhile, patients adhere to the KD were not readmitted to the hospital. Recurrence after KD discontinuation has been previously reported.^23^ It has been reported that the long‐term compliance of patients with KD is poor, at only 27–29%.^28^ KD may reduce hospital readmissions caused by recurrent convulsions. As the expert consensus recommends, KD can be tried even in the chronic phase of FIRES when convulsions are not well controlled. Influenced by classmates or friends who eat normally, it is more difficult to limit patients' carbohydrate intake when they return to school. Effective implementation of the KD is critical to prevent the recurrence of convulsions, so physicians need to emphasize the importance of maintaining dietary therapy for patients, their families, their teachers, and even their friends. The period from 2 weeks to 6 months after the initiation of the KD is called the titration phase, in which dietary adjustments are made to maximize the effects of intervention and improve the quality of life.^29^ After 3–6 months of KD, the diet and its curative effects stabilize, so this stage is known as the consolidation stage. Since the implementation of KD is relatively complex and delicate, it is of great importance to establish a KD team including specialists, nurses and dietitians. The KD team should carry out comprehensive long‐term management, and pay attention to the continuous follow‐up and dietary practices of patients, to improve the curative effect of the KD and maintain compliance as suggested.^30^ Doctors and dietitians should patiently guide patients and their families to cooperate, improve their cooking skills and their feeding methods, and timely adjust the dietary plans to ensure the normal implementation of the KD. Once patients stop the classic KD, modified Atkins diet (MAD) or low glycemic index therapy (LGIT) can be tried to achieve a curative response.

It has been reported that epilepsy and immune encephalitis secondary to viral encephalitis showed "onset‐remission‐recurrence" of bimodal course.^31^, ^32^ However, to our best knowledge, the bimodal phenomenon of FIRES was not reported previously. From the trajectory analysis, we found that 6/27 FIRES patients had bimodal disease course, increased convulsion frequency with increased body temperature. For convulsion frequency, two patients (S‐5, S‐18) whose second peak is lower than the first peak survived. But for the remaining four patients (S‐2, D‐2, D‐5, D‐7) whose second peak is higher than the first peak, only 1 case (S‐2) with severe neurological dysfunction (GOS = 3) survived. Clinically, if the bimodal course occurs and the second peak of convulsion frequency is higher, we recommend it is necessary to be alert to the possibility of poor prognosis.

Clinically, it is common that fever and infection can aggravate seizures. In our study, we found there is a linear relationship between convulsion and fever for FIRES patients. The linear relationship changed between pre‐KD and post‐KD (*R* ^2^: 0.19 vs. 0.02, slope of the trendline: 9.43 vs. 3.42) (see Figure 3). Additionally, in our study, patient S‐13 had anti‐NMDAR encephalitis on 116th day of FIRES course. Even with recurrent fever during anti‐NMDAR encephalitis, the frequency of convulsions did not increase. As can be seen from Figure 5, the slope of the trendline between the maximal body temperature and seizure frequency after KD decreased (11.99 vs. 1.74), which demonstrated the decrease of fever‐sensitivity to convulsion. Hence, we speculate that KD may reduce the fever‐sensitivity to convulsions. Additionally, we also speculate that although KD did not prevent the emergence of anti‐NMDAR encephalitis, it may help to control the convulsions of anti‐NMDAR encephalitis.

Although no specific markers were available currently, FIRES patients were characterized by cytokine abnormalities and immunotherapy effectiveness,^6^ suggesting that abnormal immunity may play a role in the onset and progression of the disease. In the study of patients with refractory epilepsy, the CSF cytokine level decreased after the KD, suggesting that this diet has immunological effects.^33^ In our study, cytokine abnormalities were observed in patient S‐14, whose IL‐8 level in CSF was high before KD and decreased after 2 weeks of KD, which paralleled the reduction in convulsions and the improvement of the patient's physical condition. Nevertheless, serum cytokines of the same patient were normal before and after KD. From the above outcomes of CSF and serum, we suggest that KD may work against FIRES through intracranial immunity.

The occurrence rate and the characteristics of imaging abnormalities of our patients were similar to those in previous reports.^2^ Four patients (S‐5, S‐9, S‐10, S‐15) in our study had normal brain imaging at the beginning of the disease and had brain atrophy during the recovery period, but they had normal cognition and returned to school. This phenomenon verified previous outcomes that patients with brain injury had a better prognosis than adults.^34^ In addition, the claustrum sign can appear in FIRES patients.^35^ Our retrospective analysis also revealed that 8 patients had the claustrum sign. In addition, we found that the feature of claustrum sign has no influence on prognosis.

Although our study has achieved some valuable results, there are still some issues that need to be further studied. For example, due to the low incidence of FIRES in the general population, the sample size was usually small, which may decrease the statistical confidence such as low *R* ^2^ suggesting a weak correlation between body temperature and convulsion frequency. At present, most FIRES studies were reported as case reports,^4^, ^5^, ^6^, ^7^ and the maximum sample size of a single center was 7 cases.^23^ Besides, to the best of our knowledge, the largest sample size reported by international multi‐centers was 77 cases in 2011.^8^ Nevertheless, no risk factors for death were identified.^8^ In our study, to decrease the influence of small sample size, we used multiple statistical methods including linear regression analysis, Pearson test, and Spearman test to analyze data and obtain consistent results such as the linear relationship between the body temperature and the seizure frequency. Besides, we made a trajectory chart for each patient and carefully observed the details of each chart to avoid omissions. Additionally, in the future, we will collect more FIRES patients to increase the sample size, which may improve our study results.

## 5 CONCLUSION

FIRES patients are critically ill with high mortality, and some may have a bimodal course. We found there may be a linear relationship between the body temperature and the convulsion frequency, and KD may reduce the fever sensitivity of convulsions. Seizure onset combined with fever, PD and KD may affect the outcome. KD can significantly reduce the convulsion frequency, reduce the application of ASM, and improve the GOS scores. Early introduction of KD may be beneficial to improve the prognosis.

## AUTHOR CONTRIBUTIONS

Li Jiang: Study Design. Juan Wang: Data Collection. Yuhang Liu: Statistical Analysis. Lingling Xie: Data Interpretation. Juan Wang, Yongfang Liu: Manuscript Preparation. Min Cheng, Lianying Feng: Literature Search. Yi Guo: Fund Collection.

## CONFLICT OF INTEREST STATEMENT

The authors declare no conflicts of interest.

## ETHICS STATEMENT

The study protocol was approved by the Institutional Review Board of the Children's Hospital of Chongqing Medical University (Approval Number: 2017‐122). The authors are accountable for all aspects of the work in ensuring that questions related to the accuracy or integrity of any part of the work are appropriately investigated and resolved.

## CONSENT FOR PUBLICATION

Written informed consent for publication was obtained from the patients' parents.

## Supporting information

Supplementary Material

Supplementary Material

Table S1

## Data Availability

The data that support the findings of this study are available on request from the corresponding author. The data are not publicly available due to privacy or ethical restrictions.
