# Supplementary material for: Clinical characteristics of 27 children with febrile infection‐related epilepsy syndrome in a single center
Source: Pediatr Discov. 2024 Jun 9;2(2):e84. doi: 10.1002/pdi3.84 (PMC12118283; doi:10.1002/pdi3.84)
Supplement: Supplementary file 1 — Supplementary Material [file PDI3-2-e84-s003.pdf]

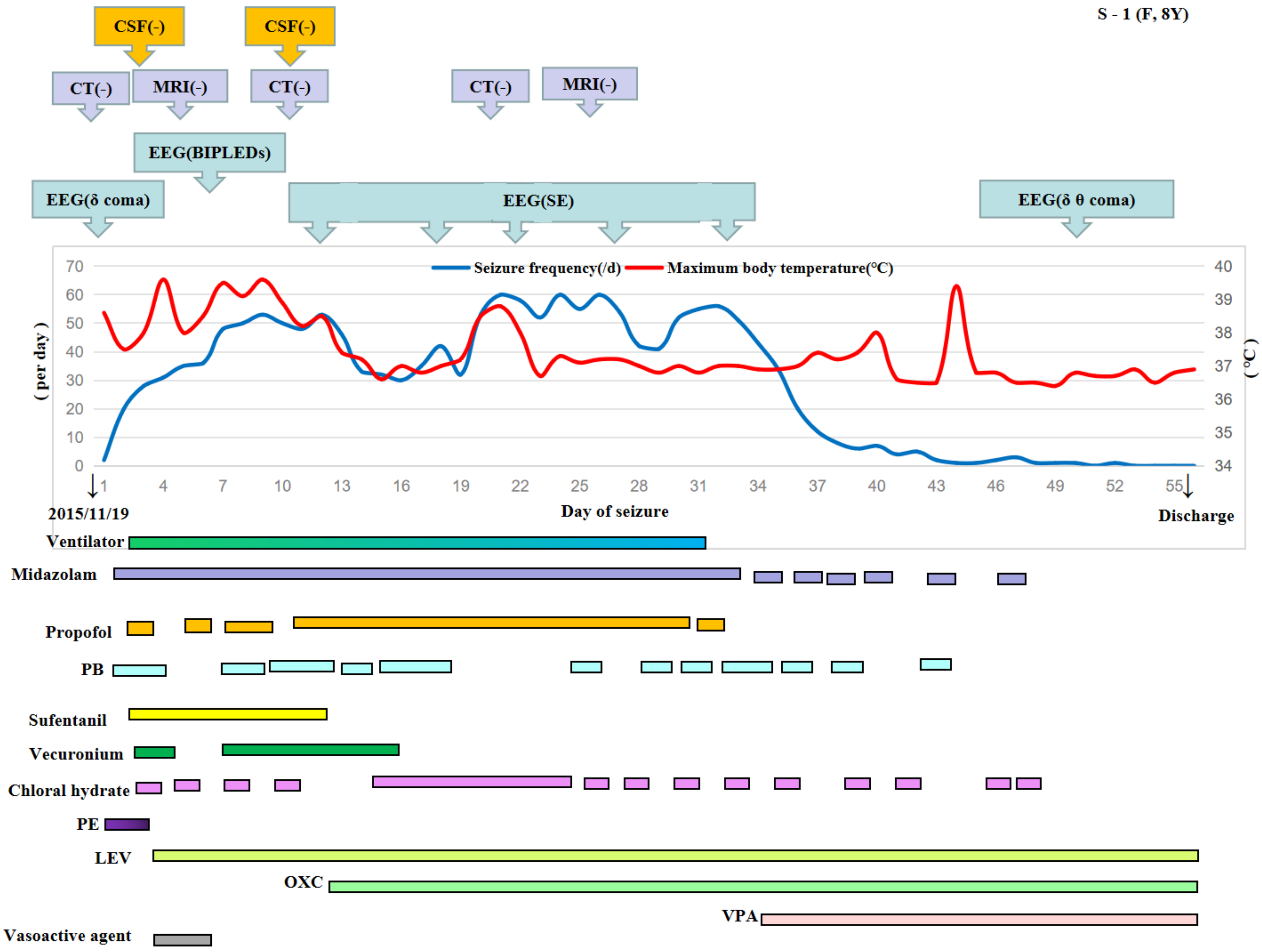

S - 1 (F, 8Y)
CSF(-)
CSF(-)
CT(-)
MRI(-)
CT(-)
CT(-)
MRI(-)
EEG(BIPLEDs)
EEG(δ coma)
EEG(SE)
EEG(δ θ coma)
Seizure frequency(/d)
Maximum body temperature(°C)
(per day)
(°C)
Day of seizure
2015/11/19
Discharge
Ventilator
Midazolam
Propofol
PB
Sufentanil
Vecuronium
Chloral hydrate
PE
LEV
OXC
VPA
Vasoactive agent

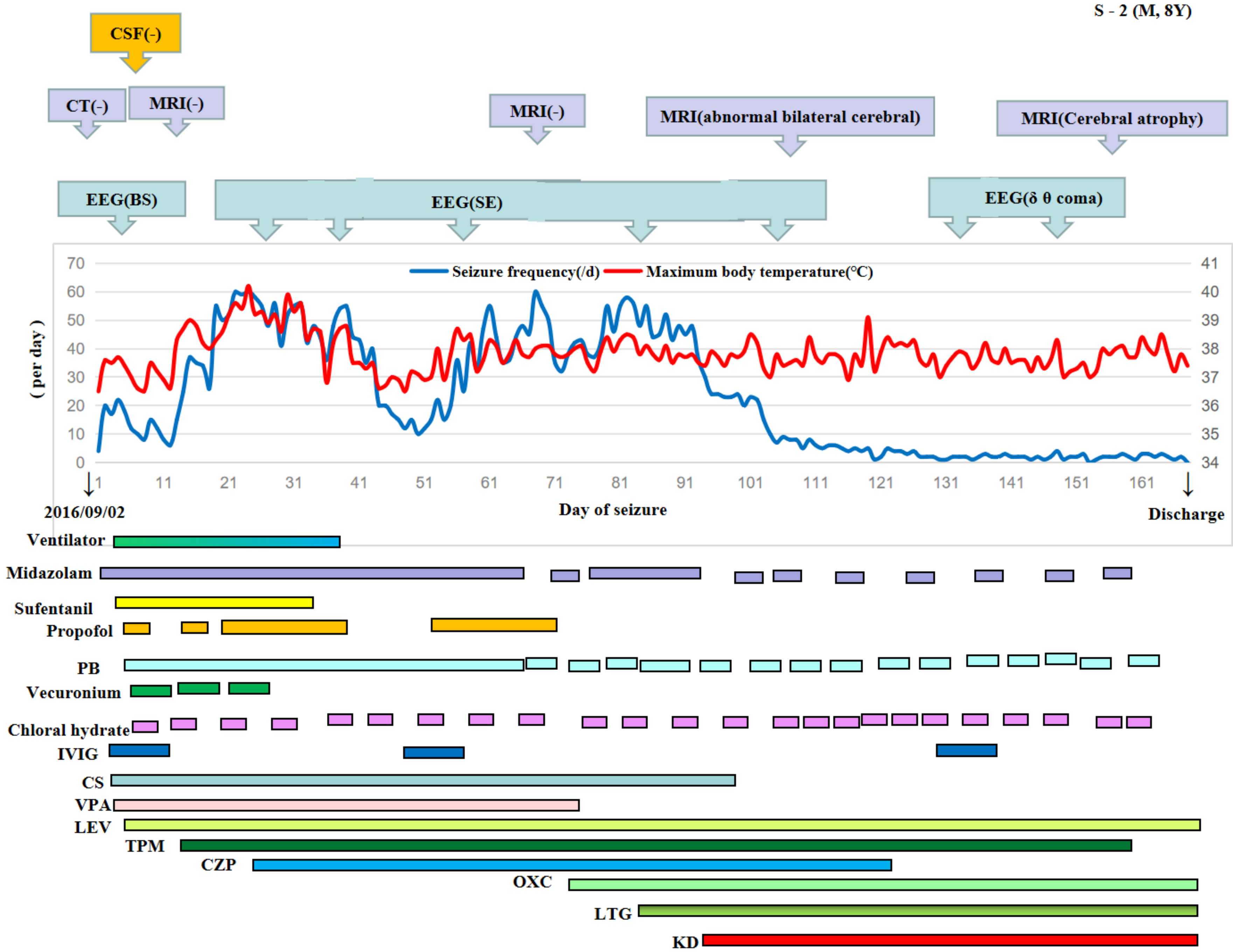

S - 2 (M, 8Y)
CSF(-)
CT(-)
MRI(-)
MRI(-)
MRI(abnormal bilateral cerebral)
MRI(Cerebral atrophy)
EEG(BS)
EEG(SE)
EEG(δ θ coma)
Seizure frequency(/d)
Maximum body temperature(°C)
(per day)
(°C)
2016/09/02
Day of seizure
Discharge
Ventilator
Midazolam
Sufentanil
Propofol
PB
Vecuronium
Chloral hydrate
IVIG
CS
VPA
LEV
TPM
CZP
OXC
LTG
KD

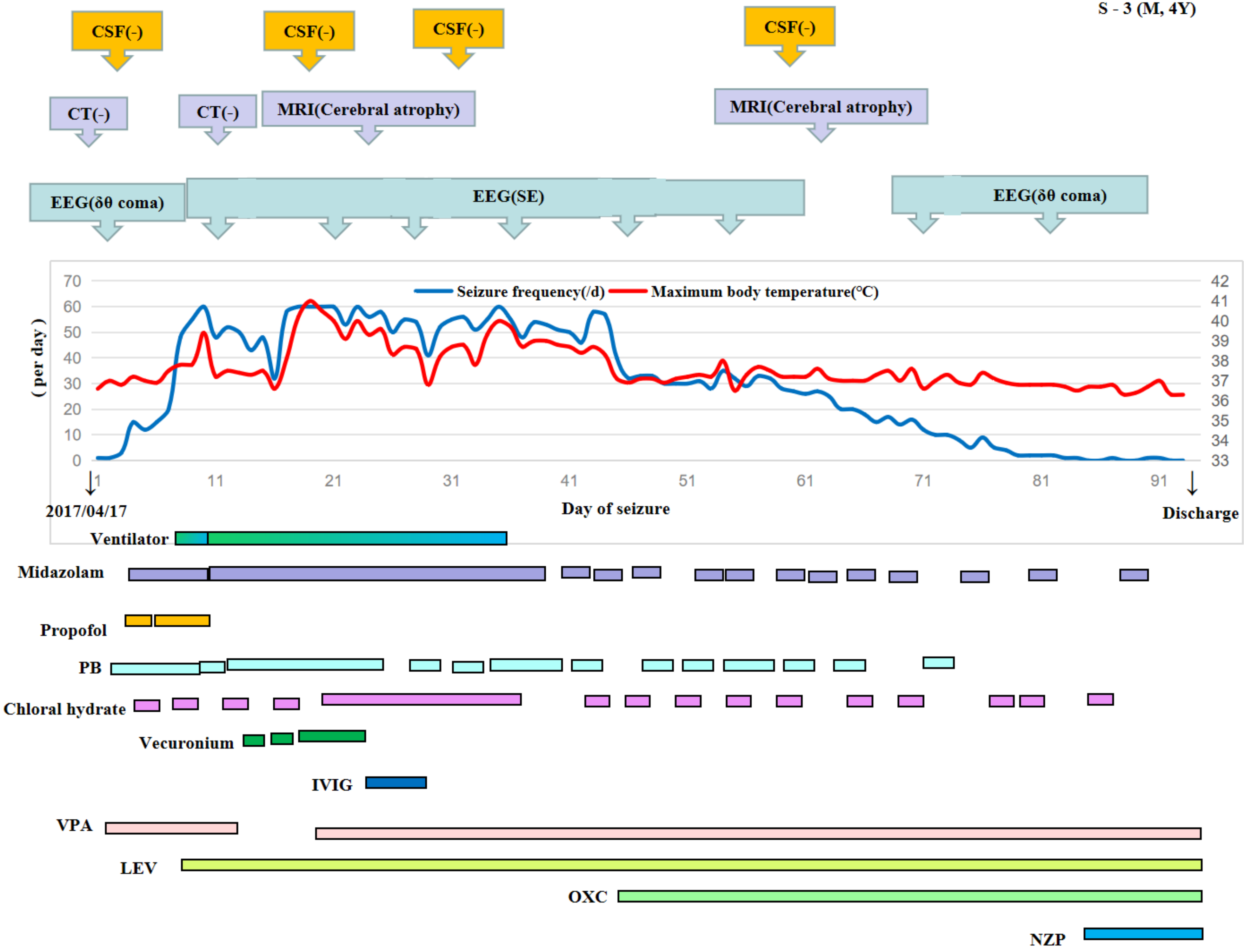
S - 3 (M, 4Y)
CSF(-)
CSF(-)
CSF(-)
CSF(-)
CT(-)
CT(-)
MRI(Cerebral atrophy)
MRI(Cerebral atrophy)
EEG(δθ coma)
EEG(SE)
EEG(δθ coma)
Seizure frequency(/d)
Maximum body temperature(℃)
(per day)
(℃)
70
60
50
40
30
20
10
0
42
41
40
39
38
37
36
35
34
33
1
11
21
31
41
51
61
71
81
91
2017/04/17
Day of seizure
Discharge
Ventilator
Midazolam
Propofol
PB
Chloral hydrate
Vecuronium
IVIG
VPA
LEV
OXC
NZP

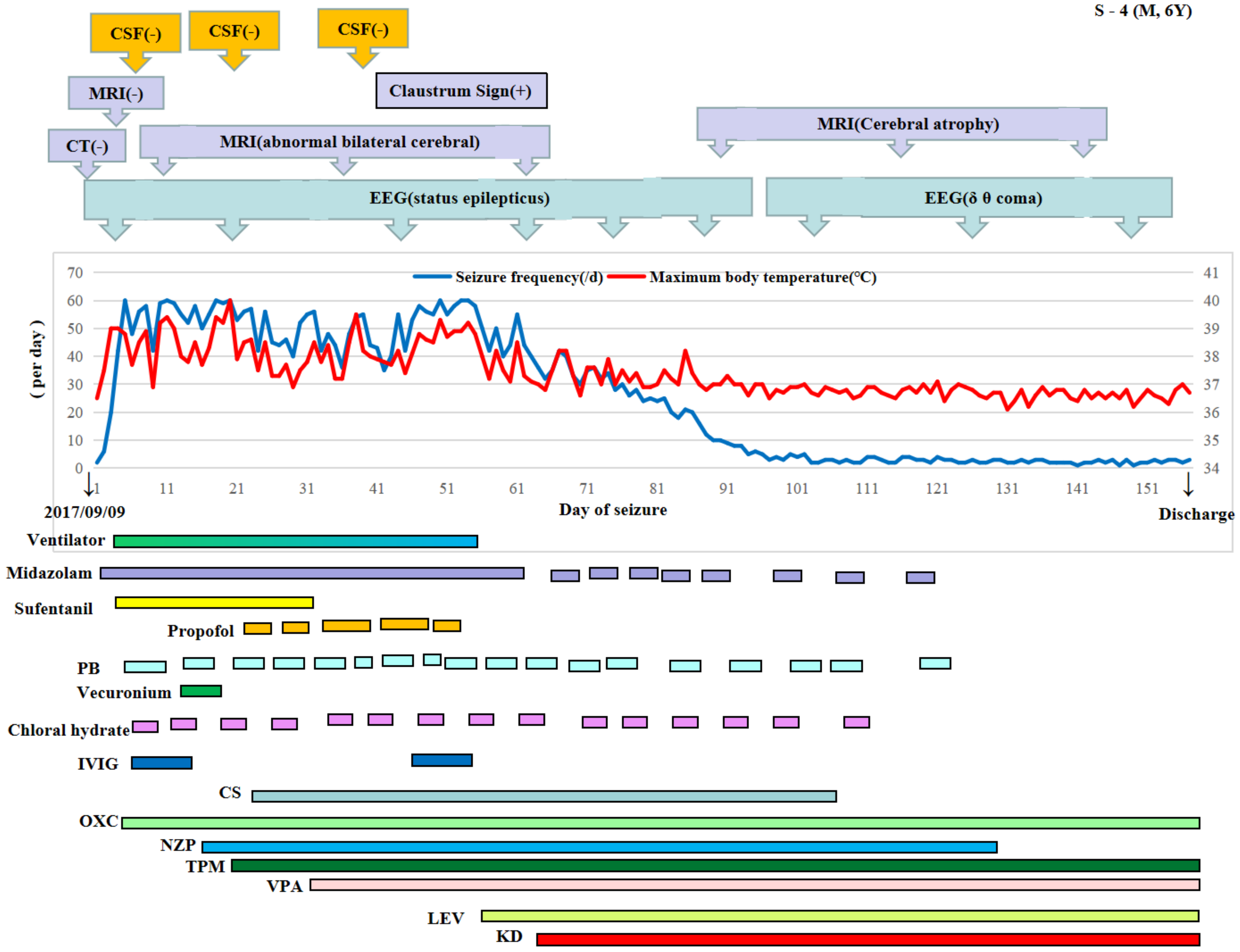

S - 4 (M, 6Y)
CSF(-)
CSF(-)
CSF(-)
MRI(-)
Claustrum Sign(+)
CT(-)
MRI(abnormal bilateral cerebral)
MRI(Cerebral atrophy)
EEG(status epilepticus)
EEG(δ θ coma)
Seizure frequency(/d)
Maximum body temperature(℃)
(per day)
(℃)
70
60
50
40
30
20
10
0
41
40
39
38
37
36
35
34
1
11
21
31
41
51
61
71
81
91
101
111
121
131
141
151
2017/09/09
Day of seizure
Discharge
Ventilator
Midazolam
Sufentanil
Propofol
PB
Vecuronium
Chloral hydrate
IVIG
CS
OXC
NZP
TPM
VPA
LEV
KD

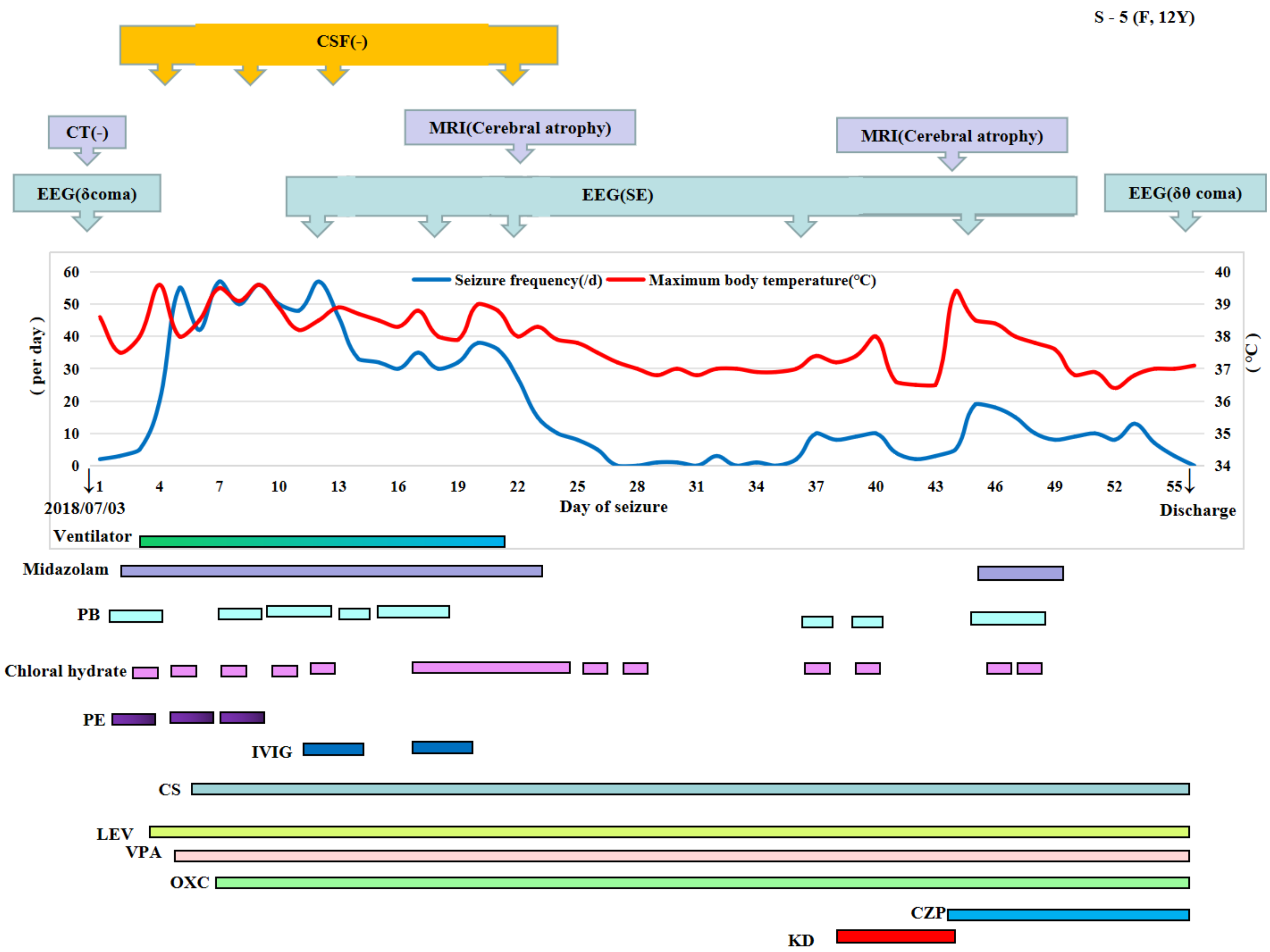
S - 5 (F, 12Y)
CSF(-)
CT(-)
MRI(Cerebral atrophy)
MRI(Cerebral atrophy)
EEG(δcoma)
EEG(SE)
EEG(δθ coma)
Seizure frequency(/d)
Maximum body temperature(°C)
(per day)
(°C)
2018/07/03
Day of seizure
Discharge
Ventilator
Midazolam
PB
Chloral hydrate
PE
IVIG
CS
LEV
VPA
OXC
CZP
KD

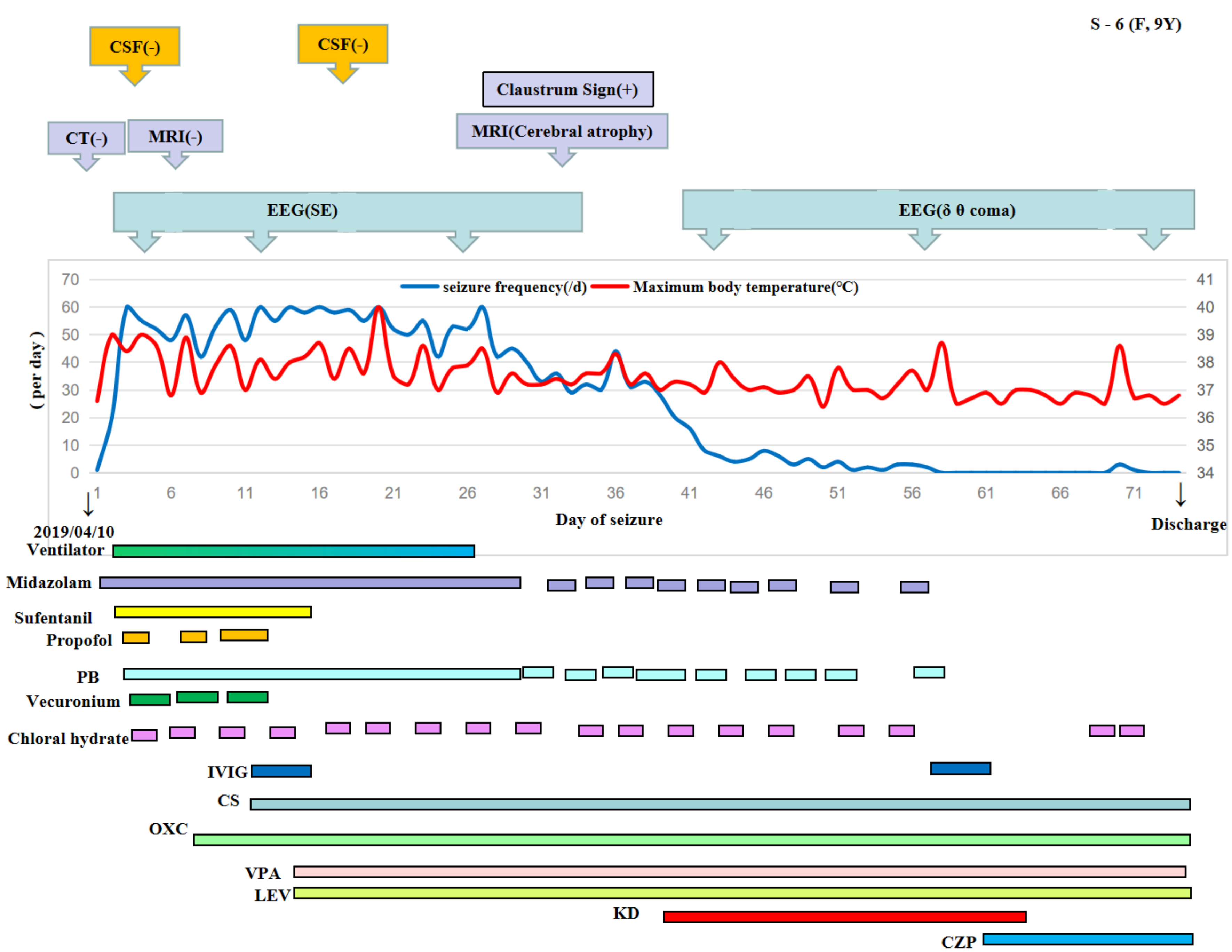

S - 6 (F, 9Y)
CSF(-)
CSF(-)
Claustrum Sign(+)
CT(-)
MRI(-)
MRI(Cerebral atrophy)
EEG(SE)
EEG(δ θ coma)
seizure frequency(/d)
Maximum body temperature(℃)
(per day)
(℃)
Day of seizure
2019/04/10
Discharge
Ventilator
Midazolam
Sufentanil
Propofol
PB
Vecuronium
Chloral hydrate
IVIG
CS
OXC
VPA
LEV
KD
CZP

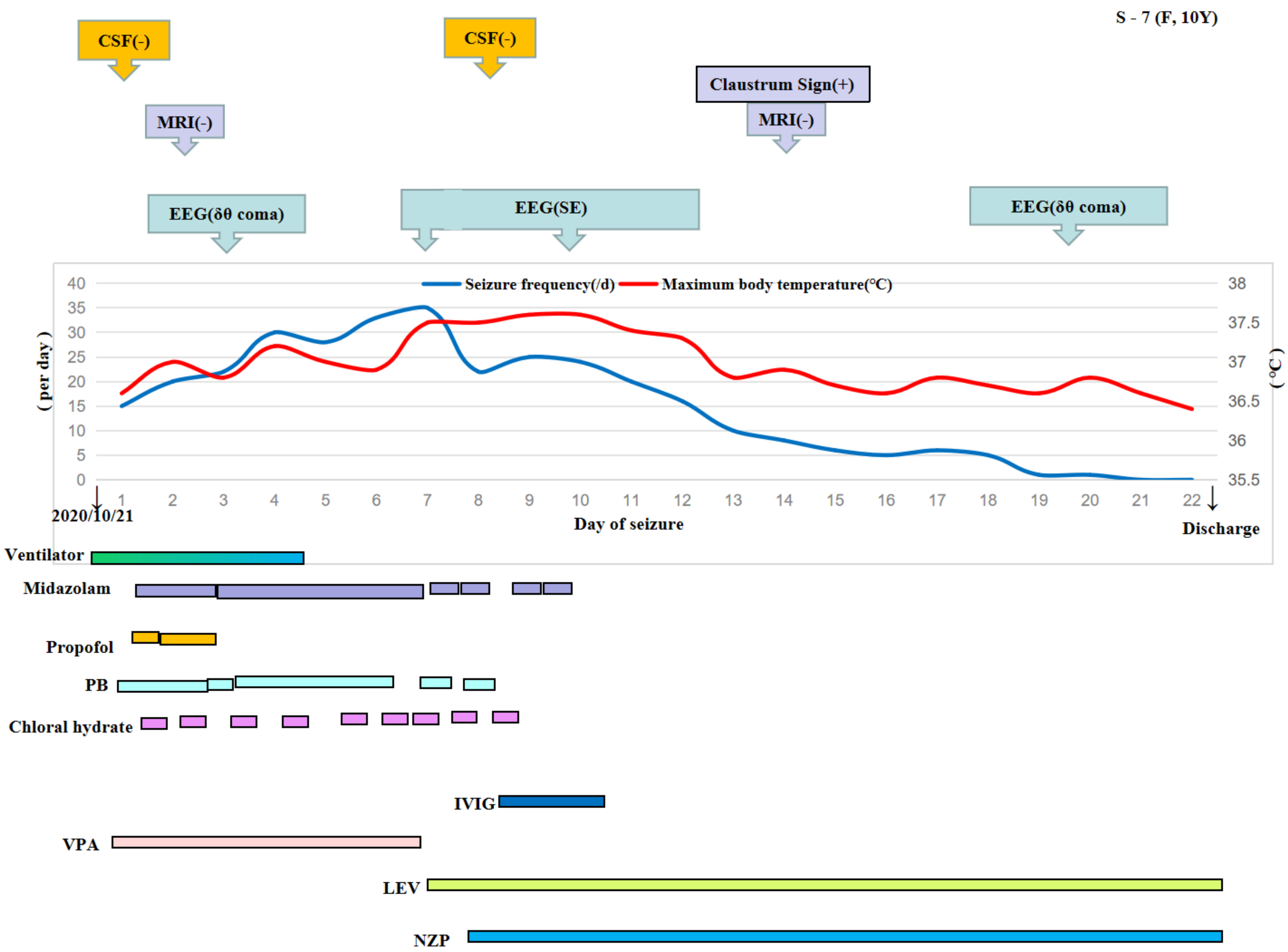
S - 7 (F, 10Y)
CSF(-)
MRI(-)
CSF(-)
Claustrum Sign(+)
MRI(-)
EEG(δθ coma)
EEG(SE)
EEG(δθ coma)
Seizure frequency(/d)
Maximum body temperature(℃)
(per day)
(℃)
2020/10/21
Day of seizure
Discharge
Ventilator
Midazolam
Propofol
PB
Chloral hydrate
IVIG
VPA
LEV
NZP

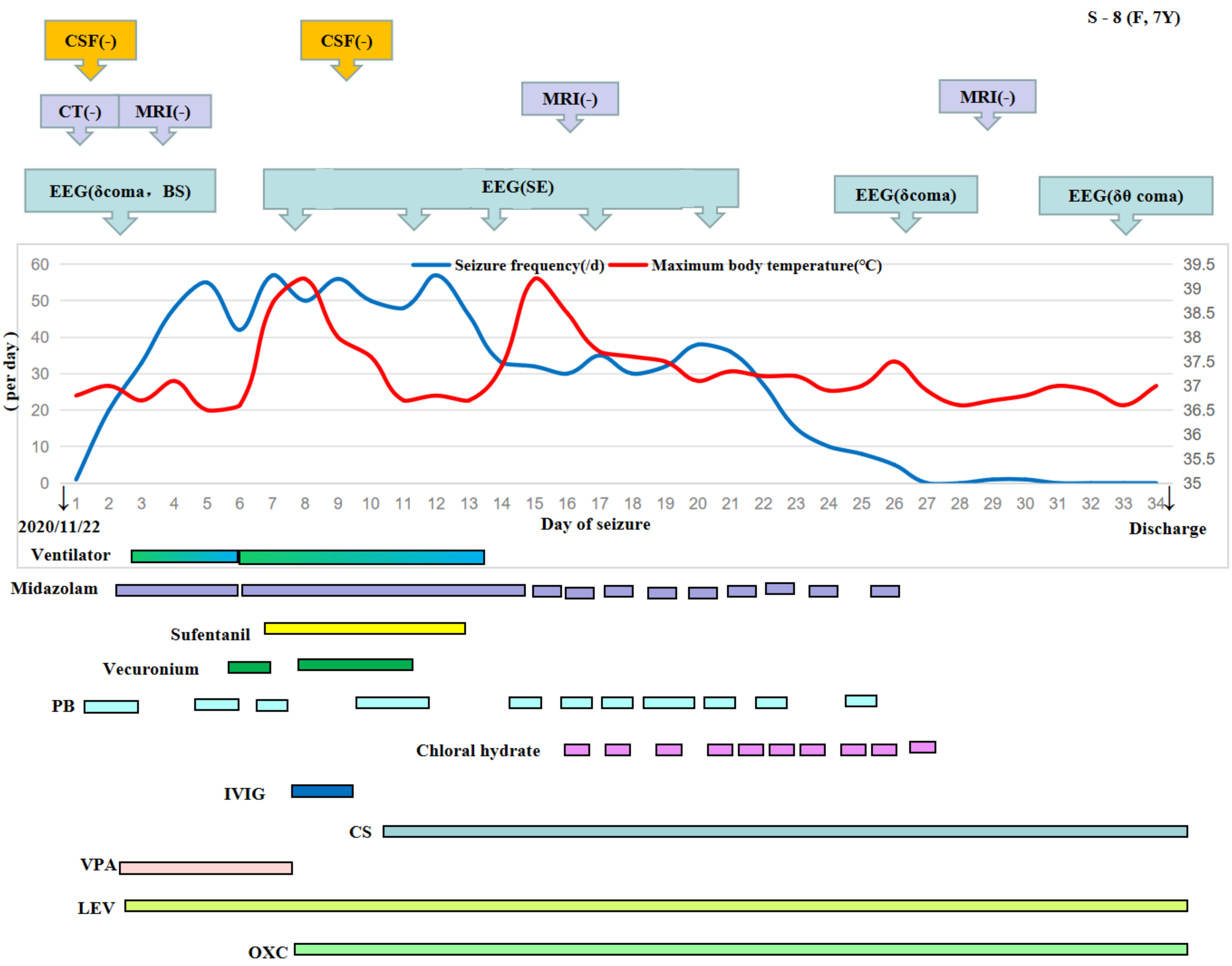
S - 8 (F, 7Y)
CSF(-)
CSF(-)
CT(-)
MRI(-)
MRI(-)
MRI(-)
EEG(δcoma，BS)
EEG(SE)
EEG(δcoma)
EEG(δθ coma)
Seizure frequency(/d)
Maximum body temperature(℃)
(per day)
(℃)
2020/11/22
Day of seizure
Discharge
Ventilator
Midazolam
Sufentanil
Vecuronium
PB
Chloral hydrate
IVIG
CS
VPA
LEV
OXC

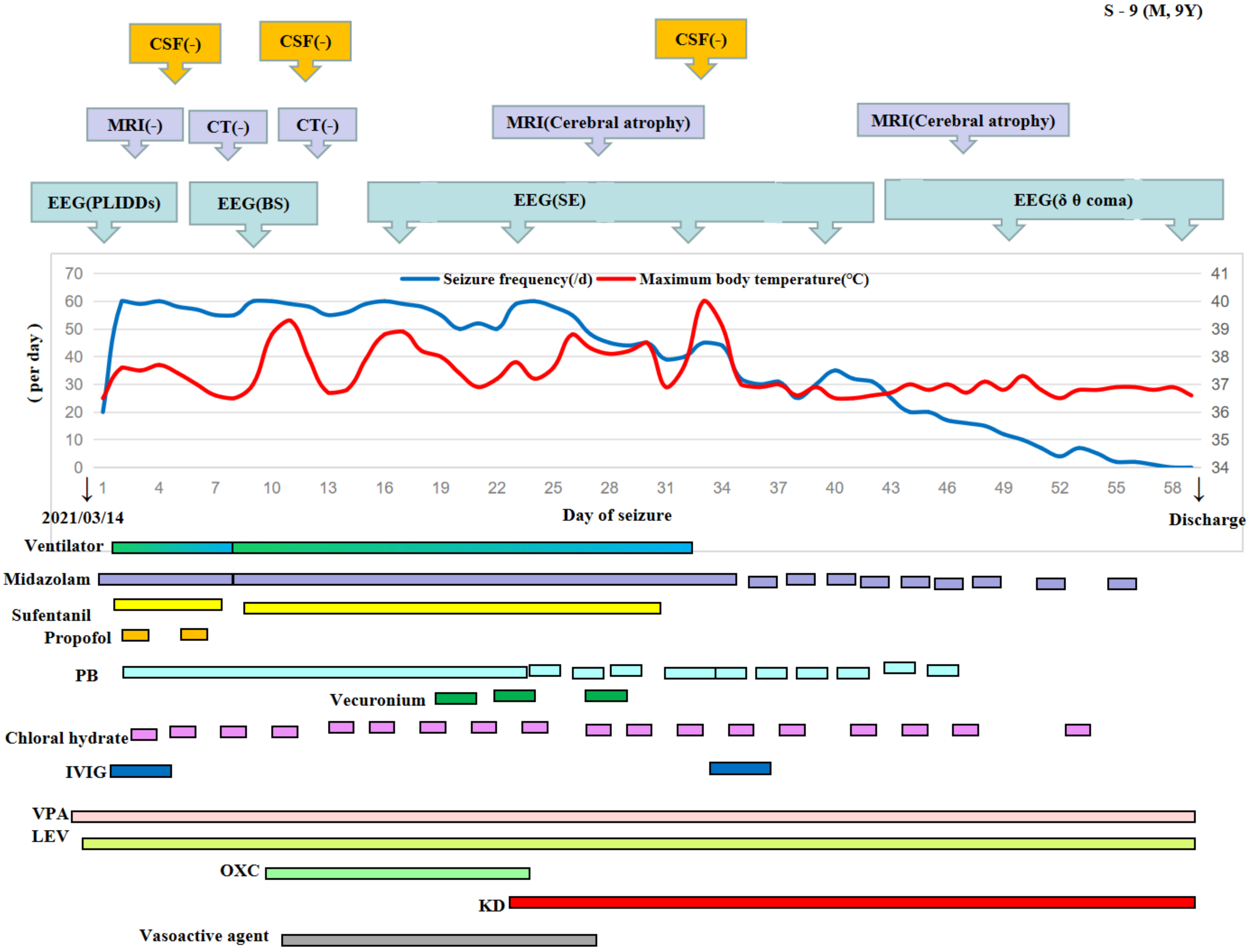

S - 9 (M, 9Y)
CSF(-)
CSF(-)
CSF(-)
MRI(-)
CT(-)
CT(-)
MRI(Cerebral atrophy)
MRI(Cerebral atrophy)
EEG(PLIDDs)
EEG(BS)
EEG(SE)
EEG(δ θ coma)
Seizure frequency(/d)
Maximum body temperature(℃)
(per day)
(℃)
70
60
50
40
30
20
10
0
41
40
39
38
37
36
35
34
1 4 7 10 13 16 19 22 25 28 31 34 37 40 43 46 49 52 55 58
2021/03/14
Day of seizure
Discharge
Ventilator
Midazolam
Sufentanil
Propofol
PB
Vecuronium
Chloral hydrate
IVIG
VPA
LEV
OXC
KD
Vasoactive agent

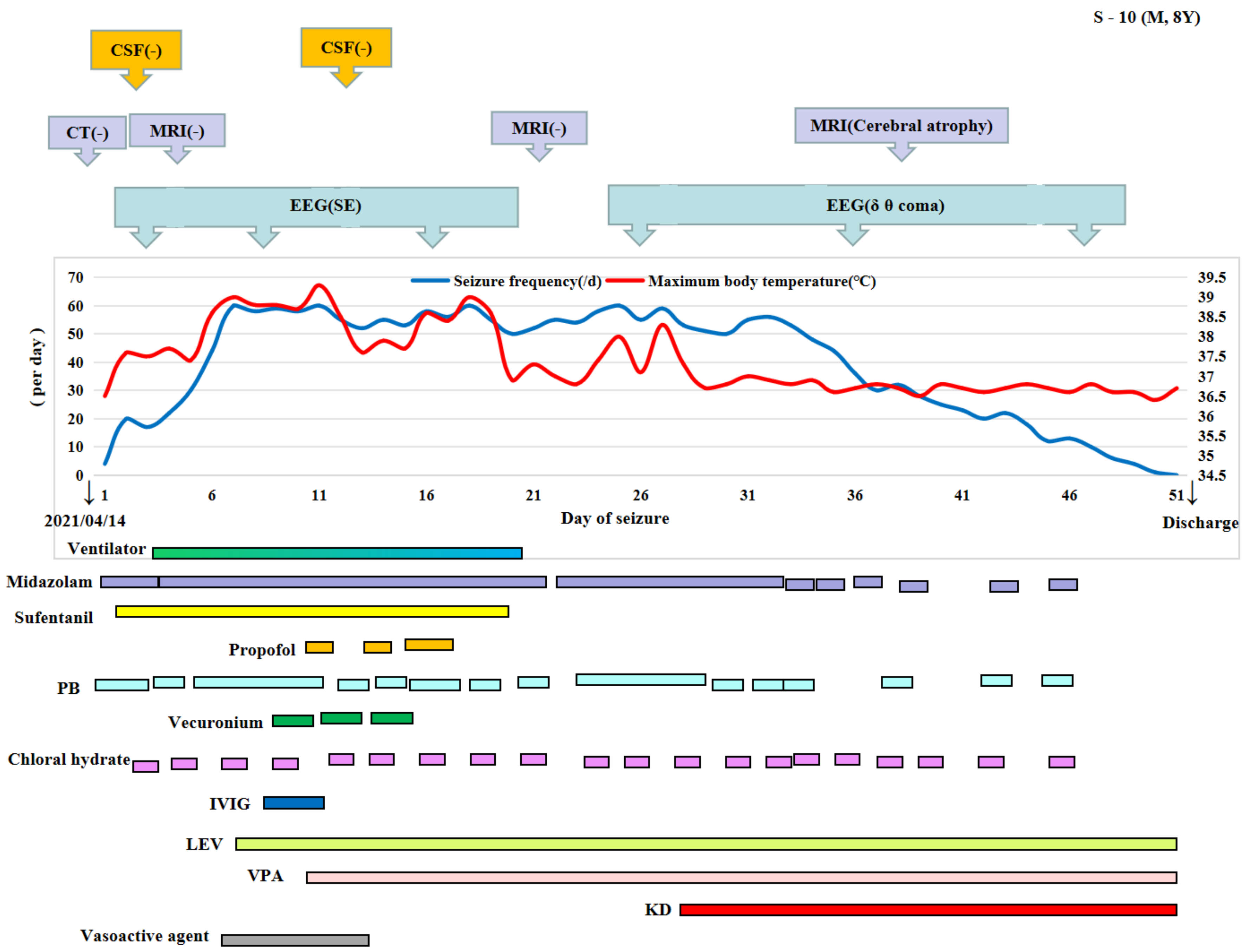
S - 10 (M, 8Y)
CSF(-)
CSF(-)
CT(-)
MRI(-)
MRI(-)
MRI(Cerebral atrophy)
EEG(SE)
EEG(δ θ coma)
Seizure frequency(/d)
Maximum body temperature(°C)
(per day)
(°C)
70
60
50
40
30
20
10
0
39.5
39
38.5
38
37.5
37
36.5
36
35.5
35
34.5
1
6
11
16
21
26
31
36
41
46
51
2021/04/14
Day of seizure
Discharge
Ventilator
Midazolam
Sufentanil
Propofol
PB
Vecuronium
Chloral hydrate
IVIG
LEV
VPA
KD
Vasoactive agent

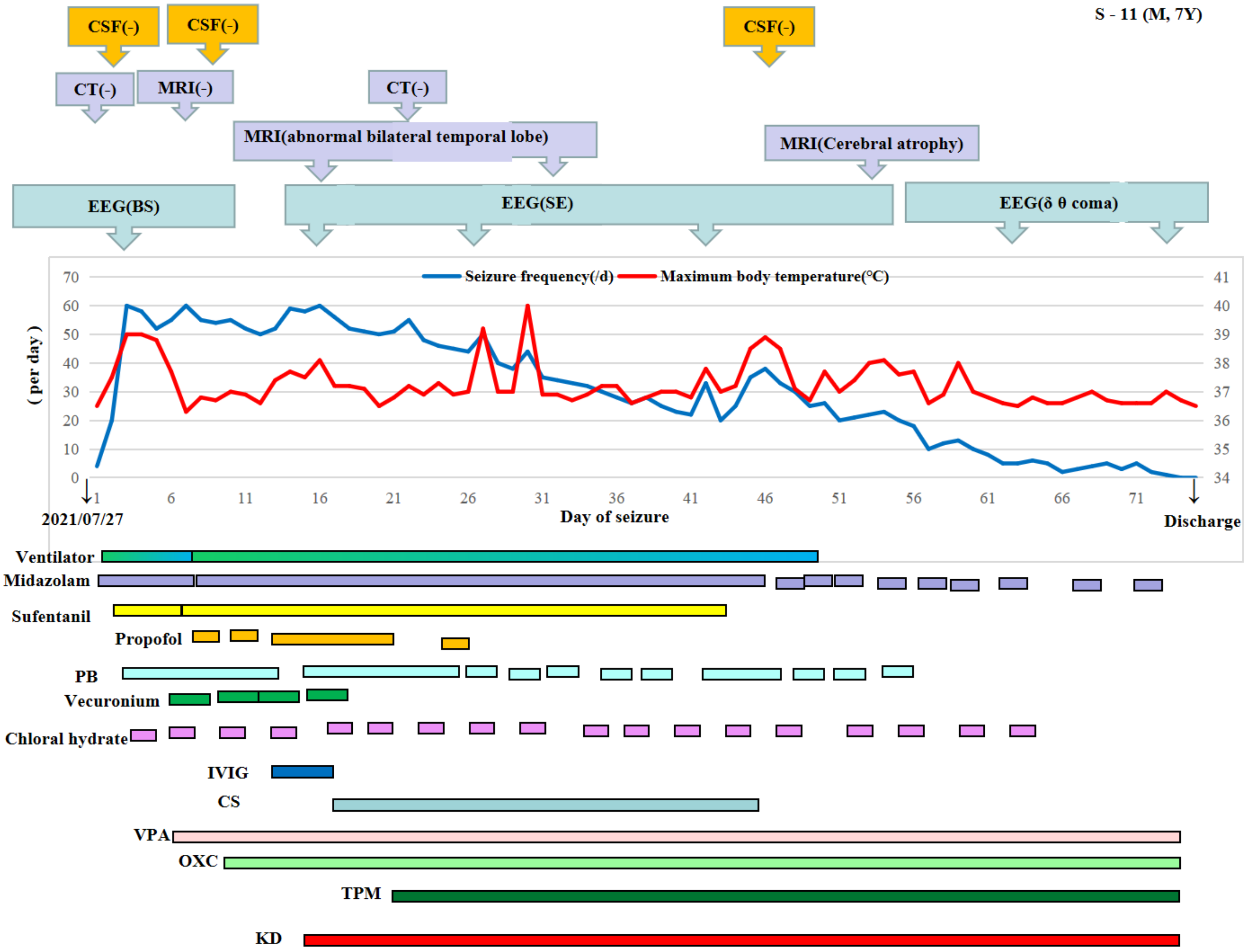

S - 11 (M, 7Y)
CSF(-)
CSF(-)
CSF(-)
CT(-)
MRI(-)
CT(-)
MRI(abnormal bilateral temporal lobe)
MRI(Cerebral atrophy)
EEG(BS)
EEG(SE)
EEG(δ θ coma)
Seizure frequency(/d)
Maximum body temperature(°C)
(per day)
(°C)
0
10
20
30
40
50
60
70
34
35
36
37
38
39
40
41
1
6
11
16
21
26
31
36
41
46
51
56
61
66
71
2021/07/27
Day of seizure
Discharge
Ventilator
Midazolam
Sufentanil
Propofol
PB
Vecuronium
Chloral hydrate
IVIG
CS
VPA
OXC
TPM
KD

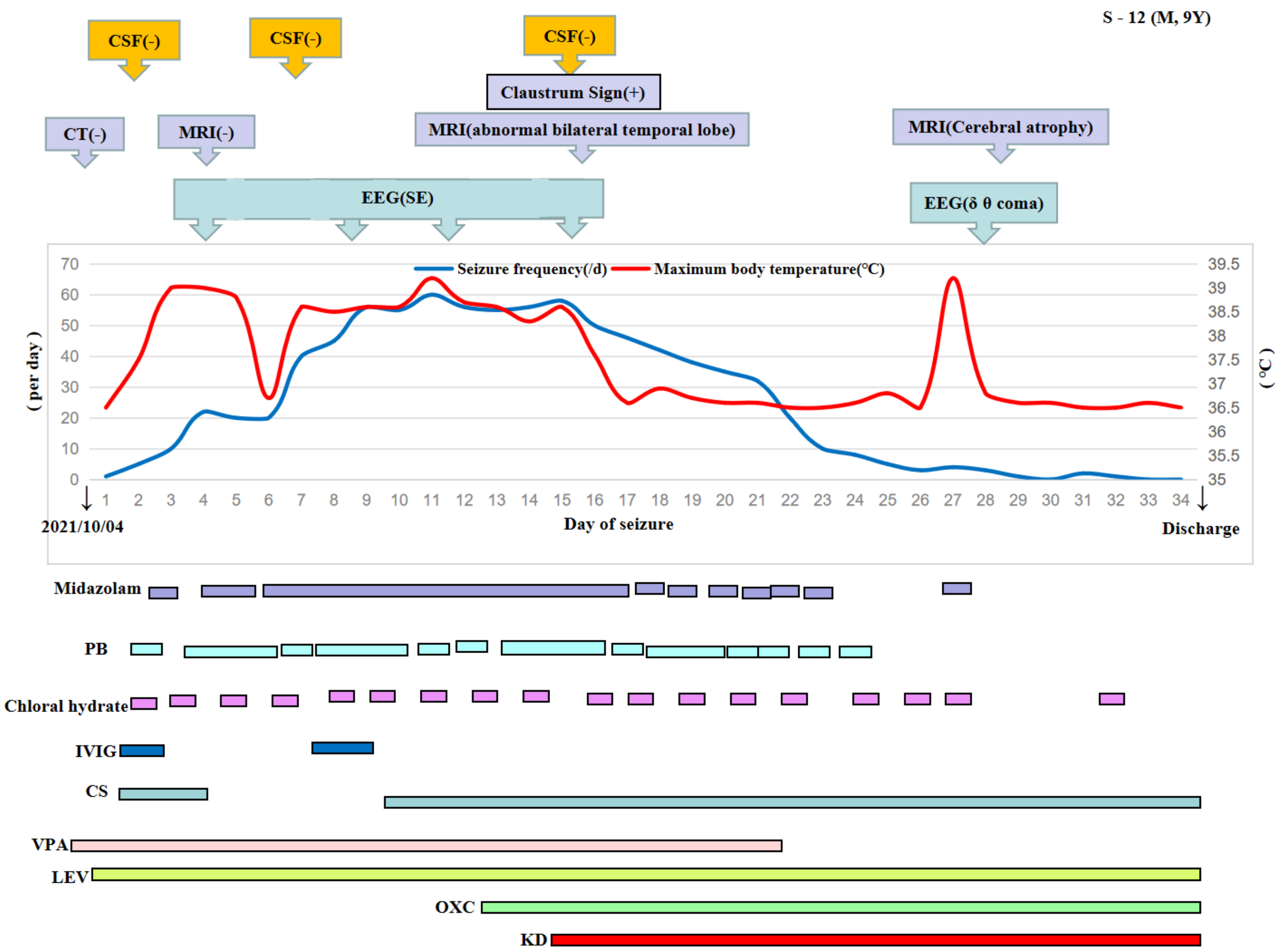
S - 12 (M, 9Y)
CSF(-)
CSF(-)
CSF(-)
Claustrum Sign(+)
CT(-)
MRI(-)
MRI(abnormal bilateral temporal lobe)
MRI(Cerebral atrophy)
EEG(SE)
EEG(δ θ coma)
Seizure frequency(/d)
Maximum body temperature(℃)
70
60
50
40
30
20
10
0
39.5
39
38.5
38
37.5
37
36.5
36
35.5
35
(per day)
(℃)
1 2 3 4 5 6 7 8 9 10 11 12 13 14 15 16 17 18 19 20 21 22 23 24 25 26 27 28 29 30 31 32 33 34
2021/10/04
Day of seizure
Discharge
Midazolam
PB
Chloral hydrate
IVIG
CS
VPA
LEV
OXC
KD

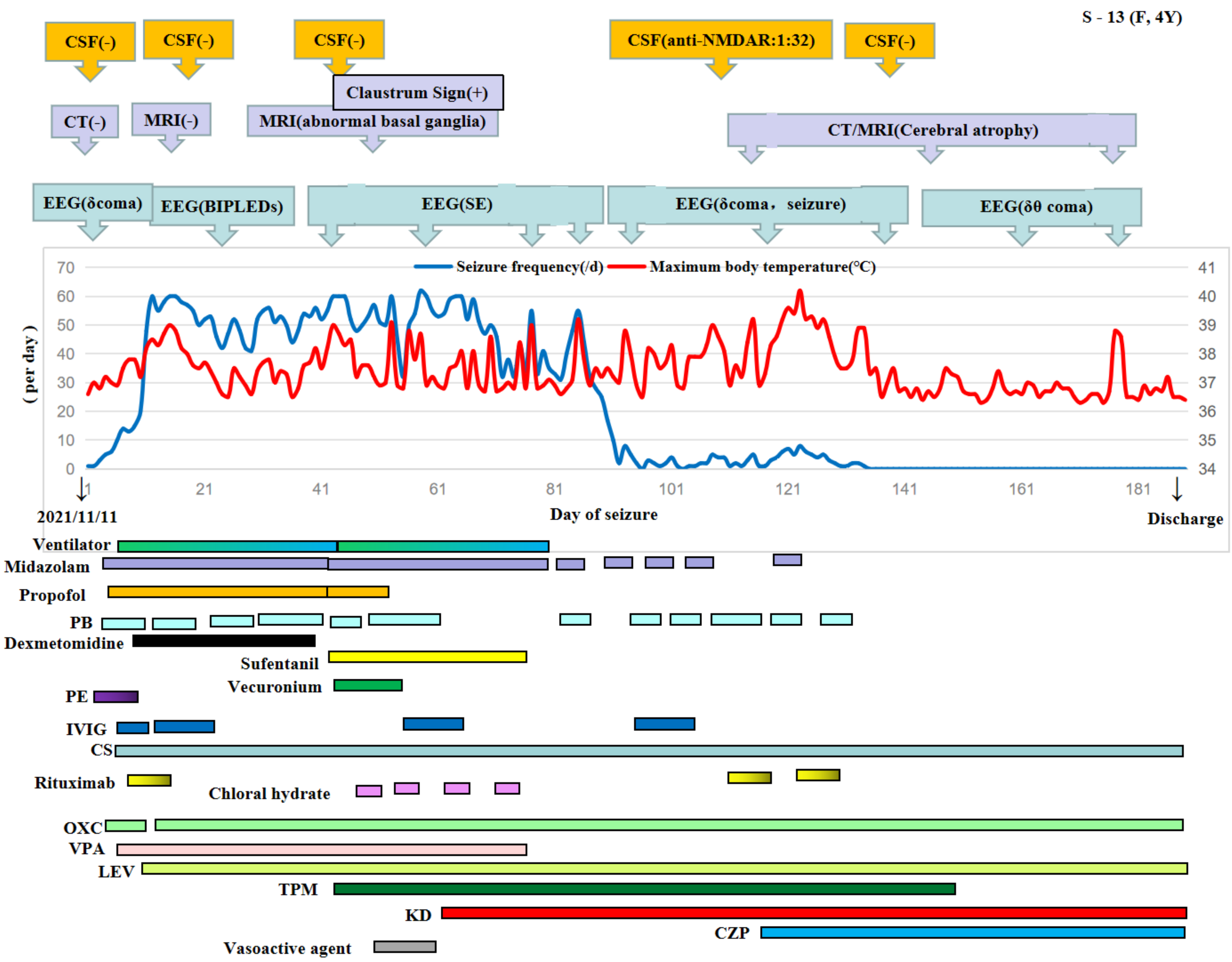

S - 13 (F, 4Y)
CSF(-)
CSF(-)
CSF(-)
CSF(anti-NMDAR:1:32)
CSF(-)
Claustrum Sign(+)
CT(-)
MRI(-)
MRI(abnormal basal ganglia)
CT/MRI(Cerebral atrophy)
EEG(δcoma)
EEG(BIPLEDs)
EEG(SE)
EEG(δcoma，seizure)
EEG(δθ coma)
Seizure frequency(/d)
Maximum body temperature(°C)
(per day)
(°C)
2021/11/11
Day of seizure
Discharge
Ventilator
Midazolam
Propofol
PB
Dexmetomidine
Sufentanil
Vecuronium
PE
IVIG
CS
Rituximab
Chloral hydrate
OXC
VPA
LEV
TPM
KD
CZP
Vasoactive agent

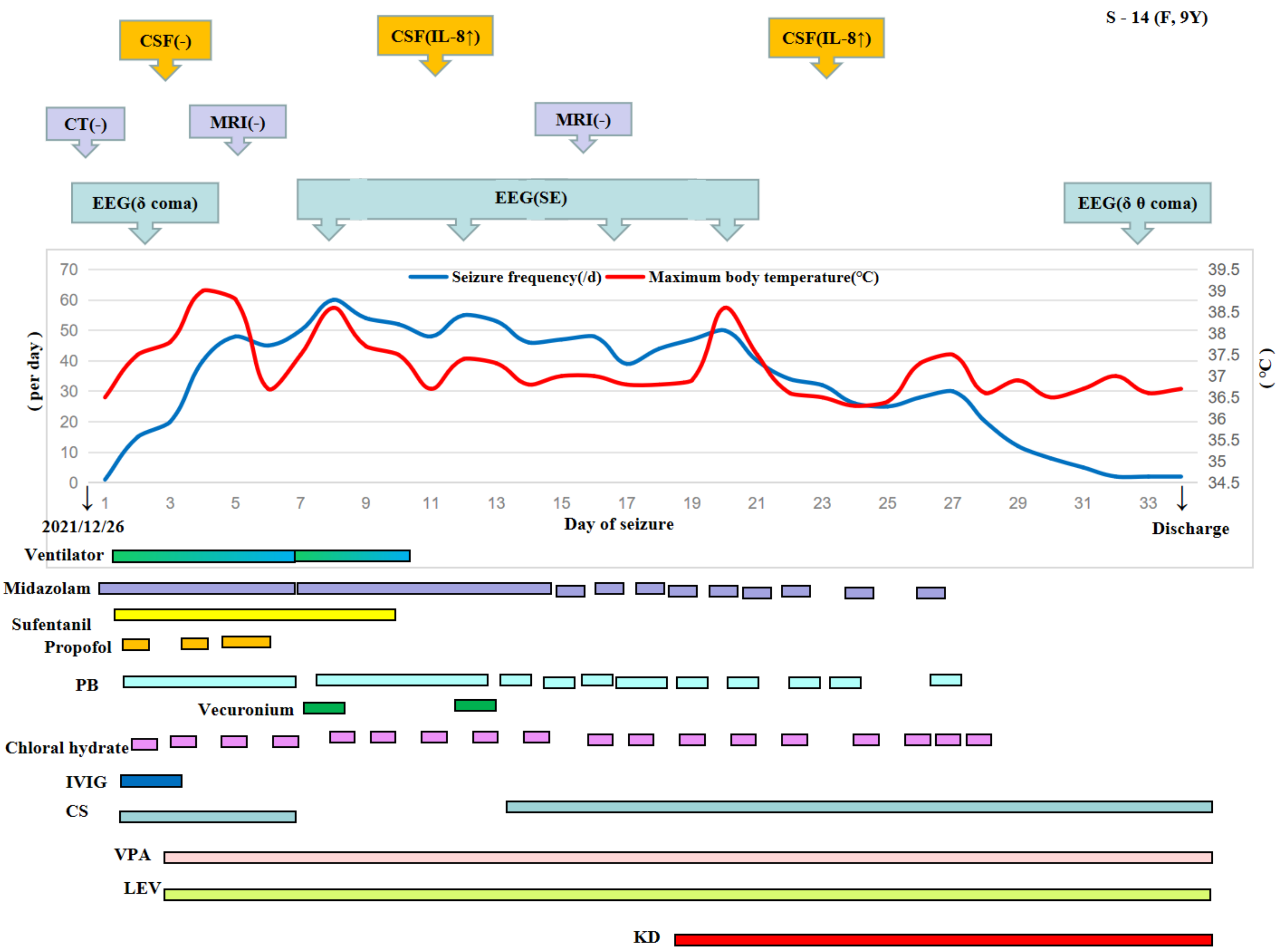

S - 14 (F, 9Y)
CSF(-)
CSF(IL-8↑)
CSF(IL-8↑)
CT(-)
MRI(-)
MRI(-)
EEG(δ coma)
EEG(SE)
EEG(δ θ coma)
Seizure frequency(/d)
Maximum body temperature(℃)
(per day)
(℃)
2021/12/26
Day of seizure
Discharge
Ventilator
Midazolam
Sufentanil
Propofol
PB
Vecuronium
Chloral hydrate
IVIG
CS
VPA
LEV
KD

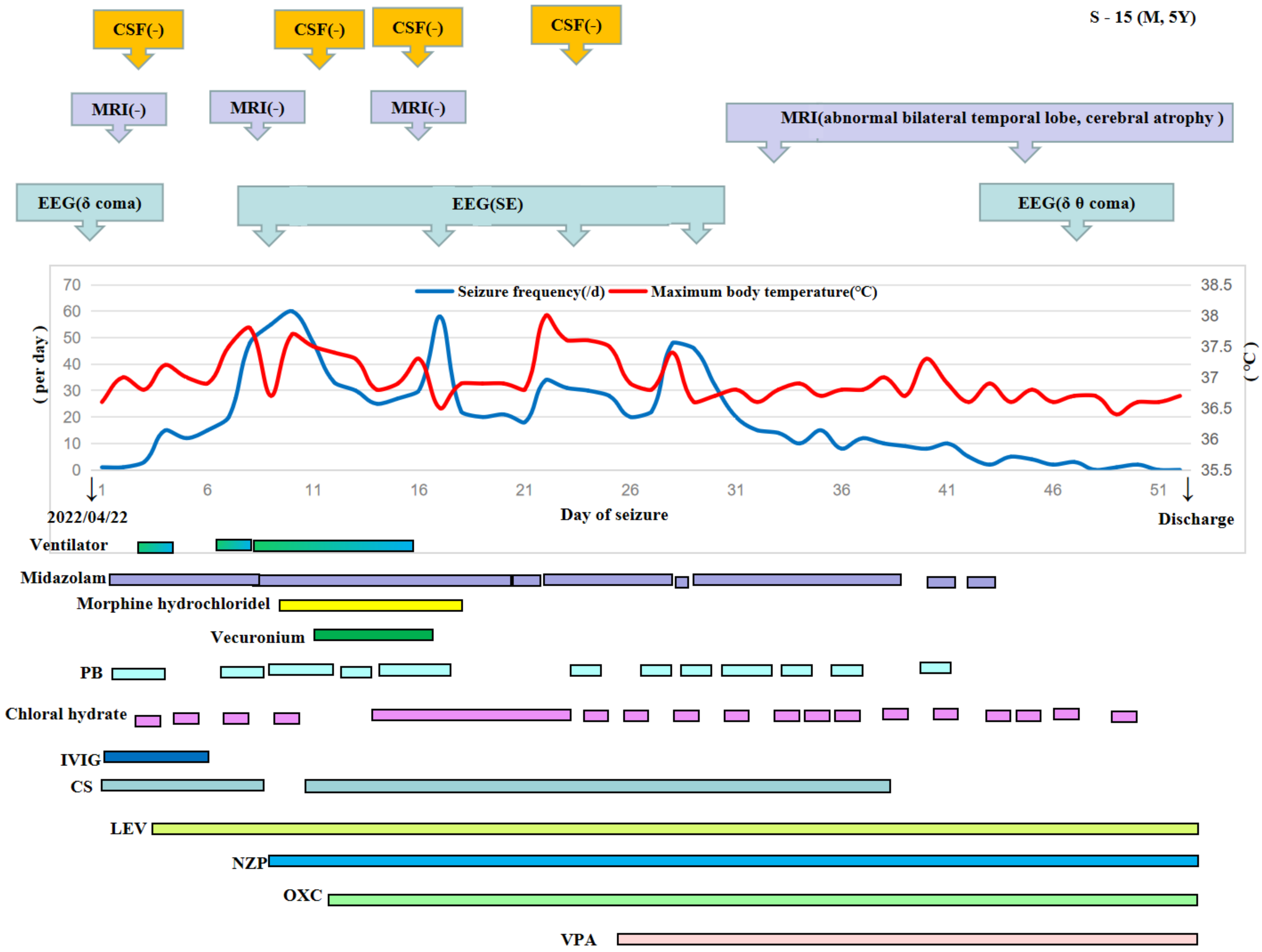
S - 15 (M, 5Y)
CSF(-)
CSF(-)
CSF(-)
CSF(-)
MRI(-)
MRI(-)
MRI(-)
MRI(abnormal bilateral temporal lobe, cerebral atrophy)
EEG(δ coma)
EEG(SE)
EEG(δ θ coma)
Seizure frequency(/d)
Maximum body temperature(°C)
(per day)
(°C)
0
10
20
30
40
50
60
70
35.5
36
36.5
37
37.5
38
38.5
1
6
11
16
21
26
31
36
41
46
51
2022/04/22
Day of seizure
Discharge
Ventilator
Midazolam
Morphine hydrochloridel
Vecuronium
PB
Chloral hydrate
IVIG
CS
LEV
NZP
OXC
VPA

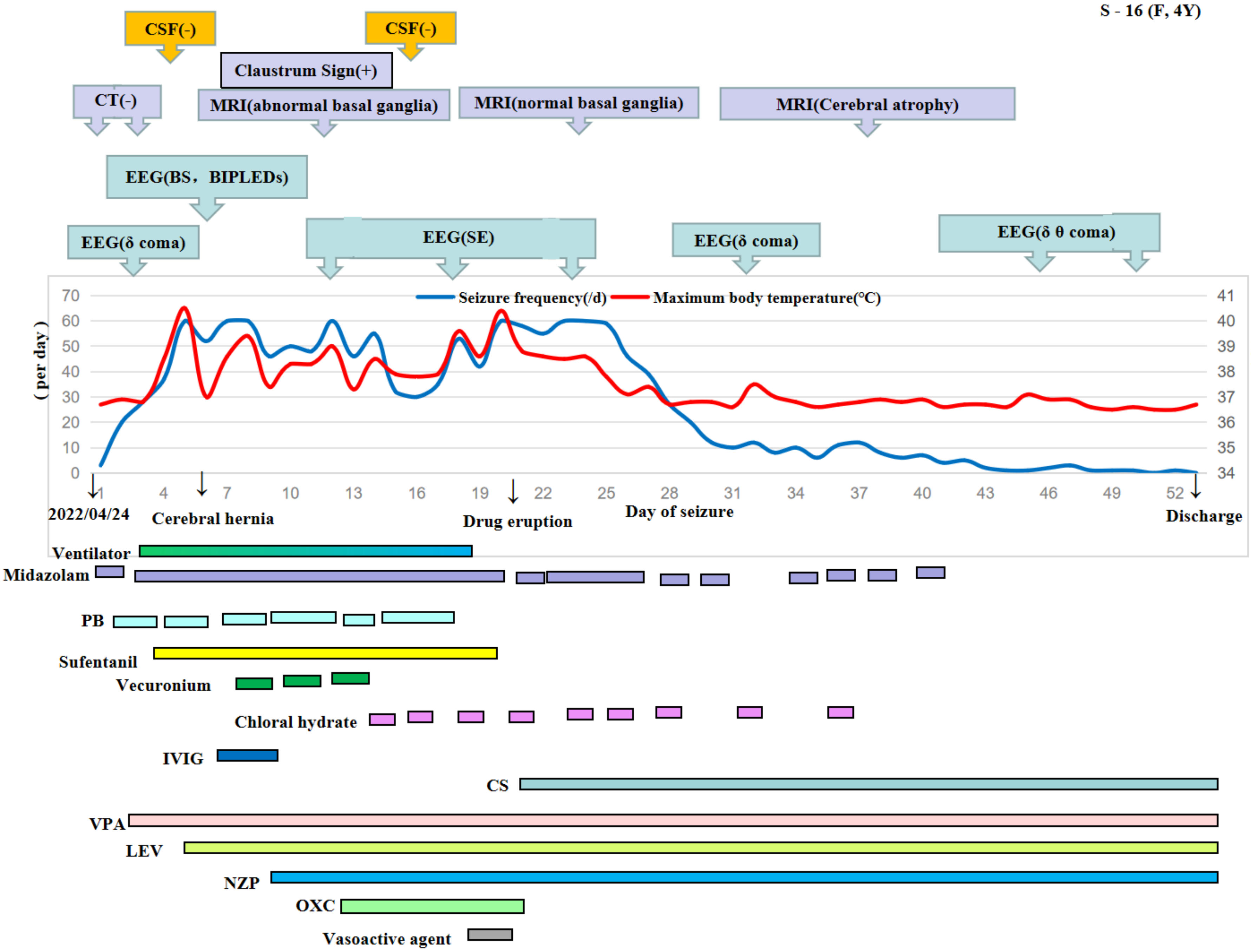

S - 16 (F, 4Y)
CSF(-)
CSF(-)
Claustrum Sign(+)
CT(-)
MRI(abnormal basal ganglia)
MRI(normal basal ganglia)
MRI(Cerebral atrophy)
EEG(BS，BIPLEDs)
EEG(δ coma)
EEG(SE)
EEG(δ coma)
EEG(δ θ coma)
Seizure frequency(/d)
Maximum body temperature(℃)
(per day)
(℃)
2022/04/24
Cerebral hernia
Drug eruption
Day of seizure
Discharge
Ventilator
Midazolam
PB
Sufentanil
Vecuronium
Chloral hydrate
IVIG
CS
VPA
LEV
NZP
OXC
Vasoactive agent

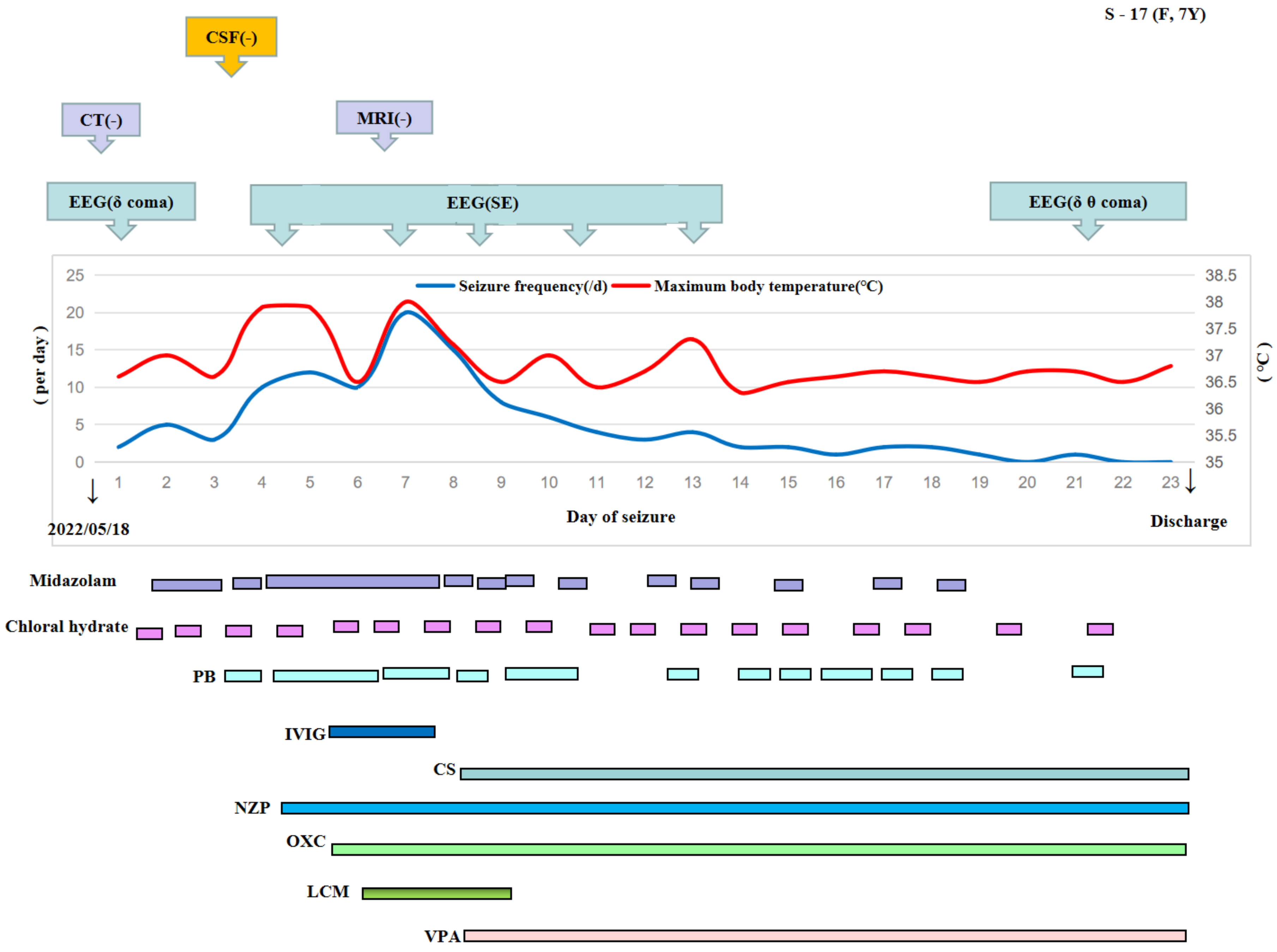

S - 17 (F, 7Y)
CSF(-)
CT(-)
MRI(-)
EEG(δ coma)
EEG(SE)
EEG(δ θ coma)
Seizure frequency(/d)
Maximum body temperature(°C)
(per day)
(°C)
2022/05/18
Day of seizure
Discharge
Midazolam
Chloral hydrate
PB
IVIG
CS
NZP
OXC
LCM
VPA

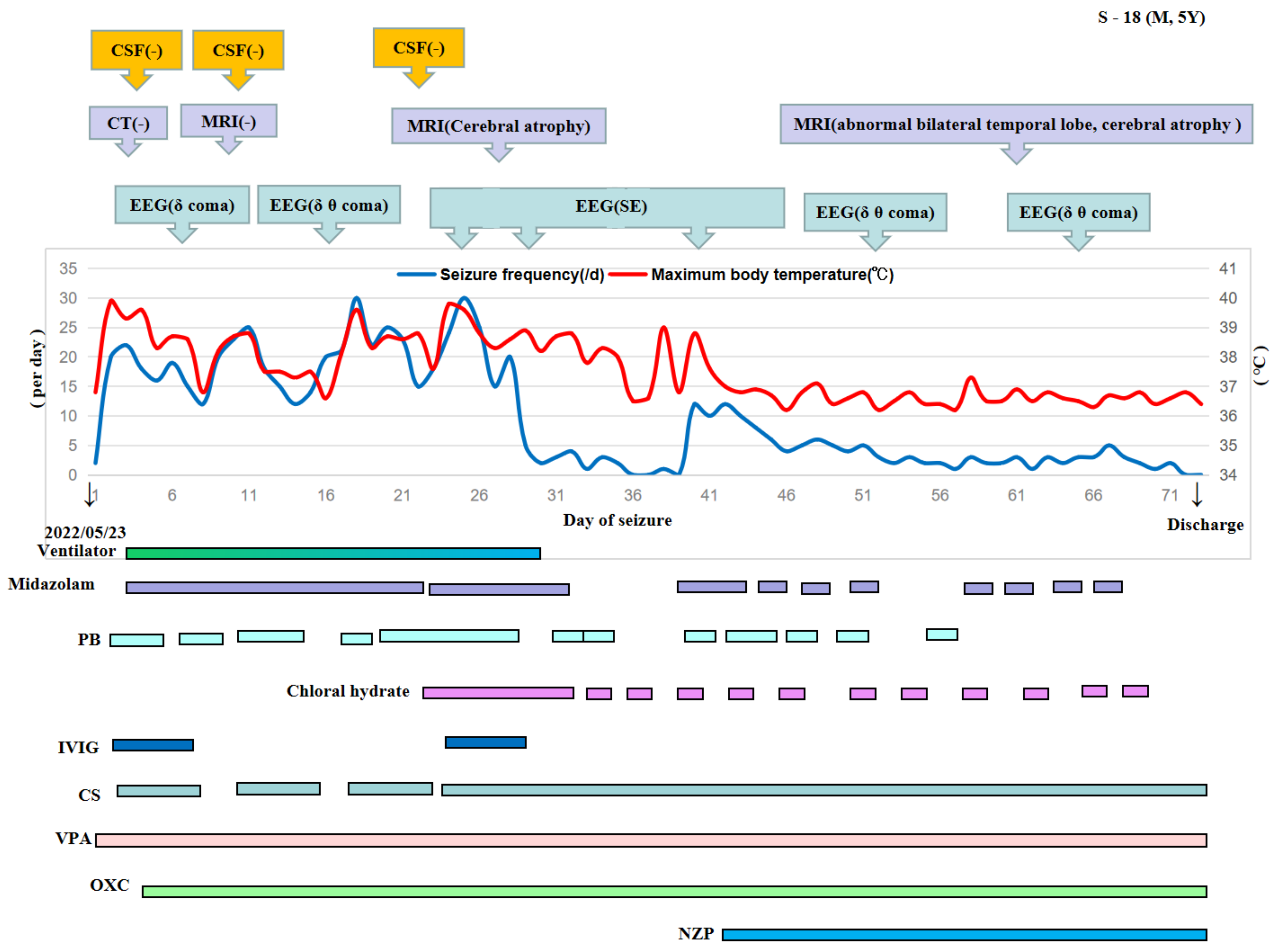
S - 18 (M, 5Y)
CSF(-)
CSF(-)
CSF(-)
CT(-)
MRI(-)
MRI(Cerebral atrophy)
MRI(abnormal bilateral temporal lobe, cerebral atrophy)
EEG(δ coma)
EEG(δ θ coma)
EEG(SE)
EEG(δ θ coma)
EEG(δ θ coma)
Seizure frequency(/d)
Maximum body temperature(℃)
(per day)
(℃)
Day of seizure
2022/05/23
Discharge
Ventilator
Midazolam
PB
Chloral hydrate
IVIG
CS
VPA
OXC
NZP

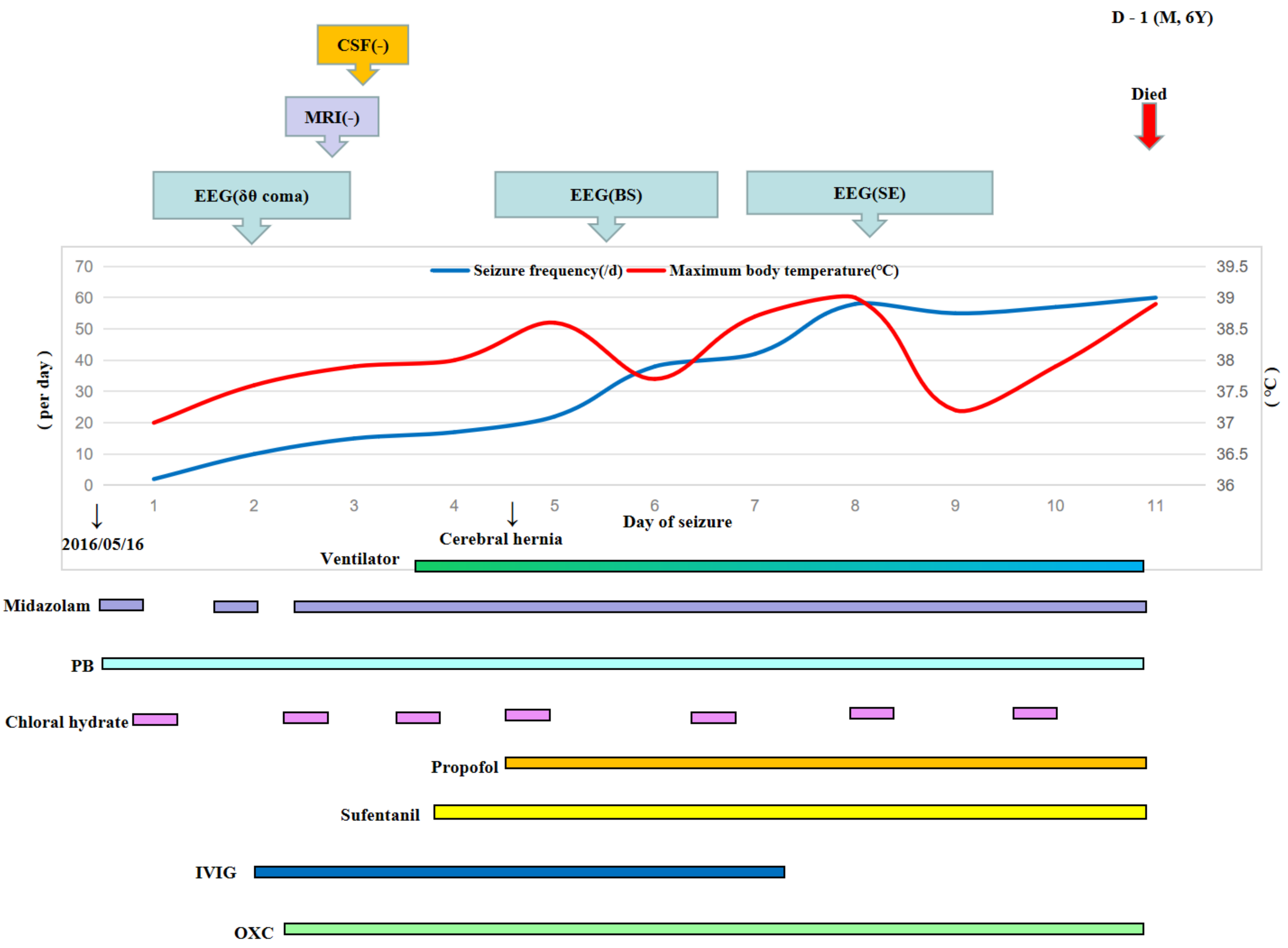

D - 1 (M, 6Y)
CSF(-)
MRI(-)
EEG(δθ coma)
EEG(BS)
EEG(SE)
Died
Seizure frequency(/d)
Maximum body temperature(℃)
(per day)
(℃)
70
60
50
40
30
20
10
0
39.5
39
38.5
38
37.5
37
36.5
36
1
2
3
4
5
6
7
8
9
10
11
Day of seizure
2016/05/16
Cerebral hernia
Ventilator
Midazolam
PB
Chloral hydrate
Propofol
Sufentanil
IVIG
OXC

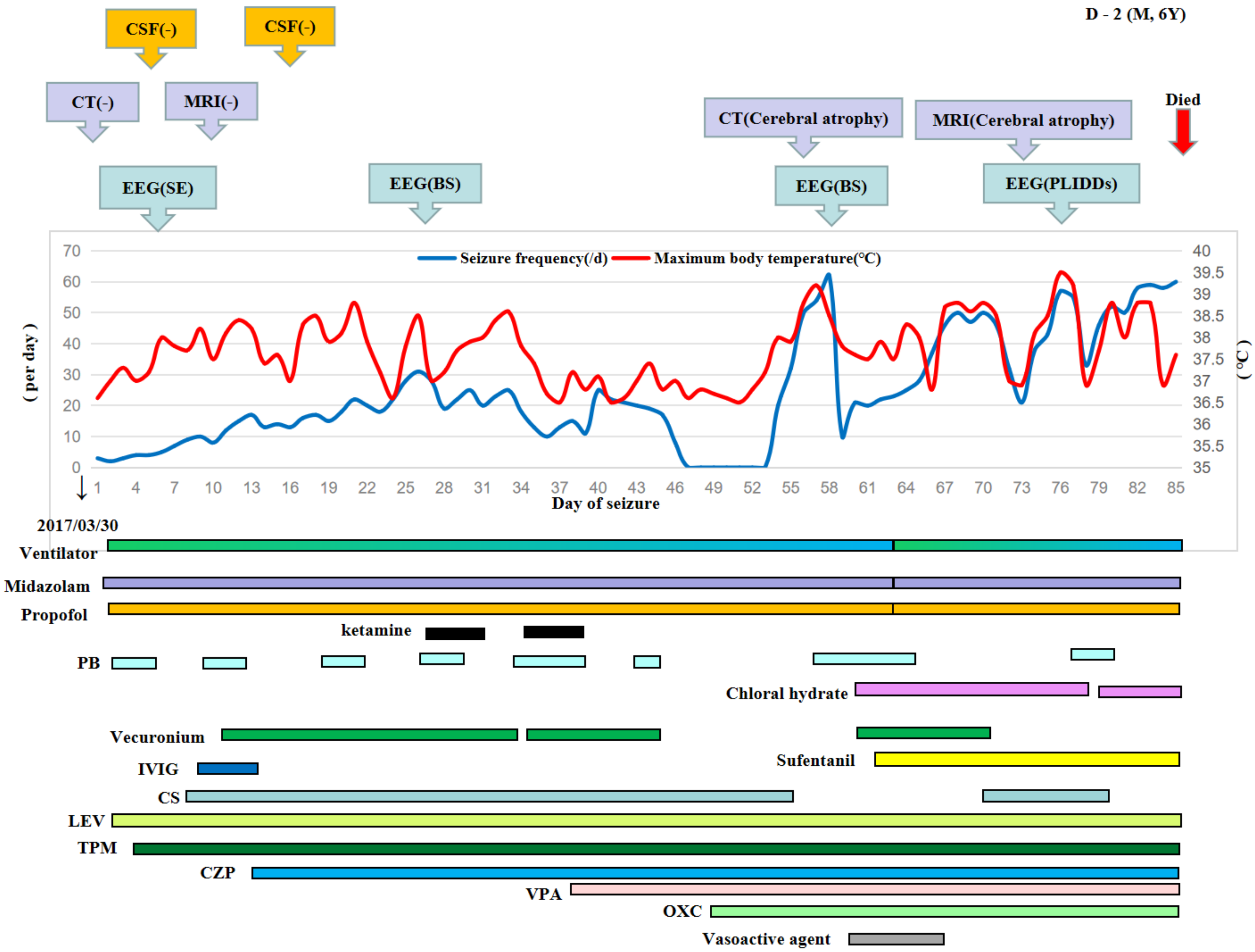

D - 2 (M, 6Y)
CSF(-)
CSF(-)
CT(-)
MRI(-)
CT(Cerebral atrophy)
MRI(Cerebral atrophy)
Died
EEG(SE)
EEG(BS)
EEG(BS)
EEG(PLIDDs)
Seizure frequency(/d)
Maximum body temperature(°C)
(per day)
(°C)
Day of seizure
2017/03/30
Ventilator
Midazolam
Propofol
ketamine
PB
Chloral hydrate
Vecuronium
IVIG
Sufentanil
CS
LEV
TPM
CZP
VPA
OXC
Vasoactive agent

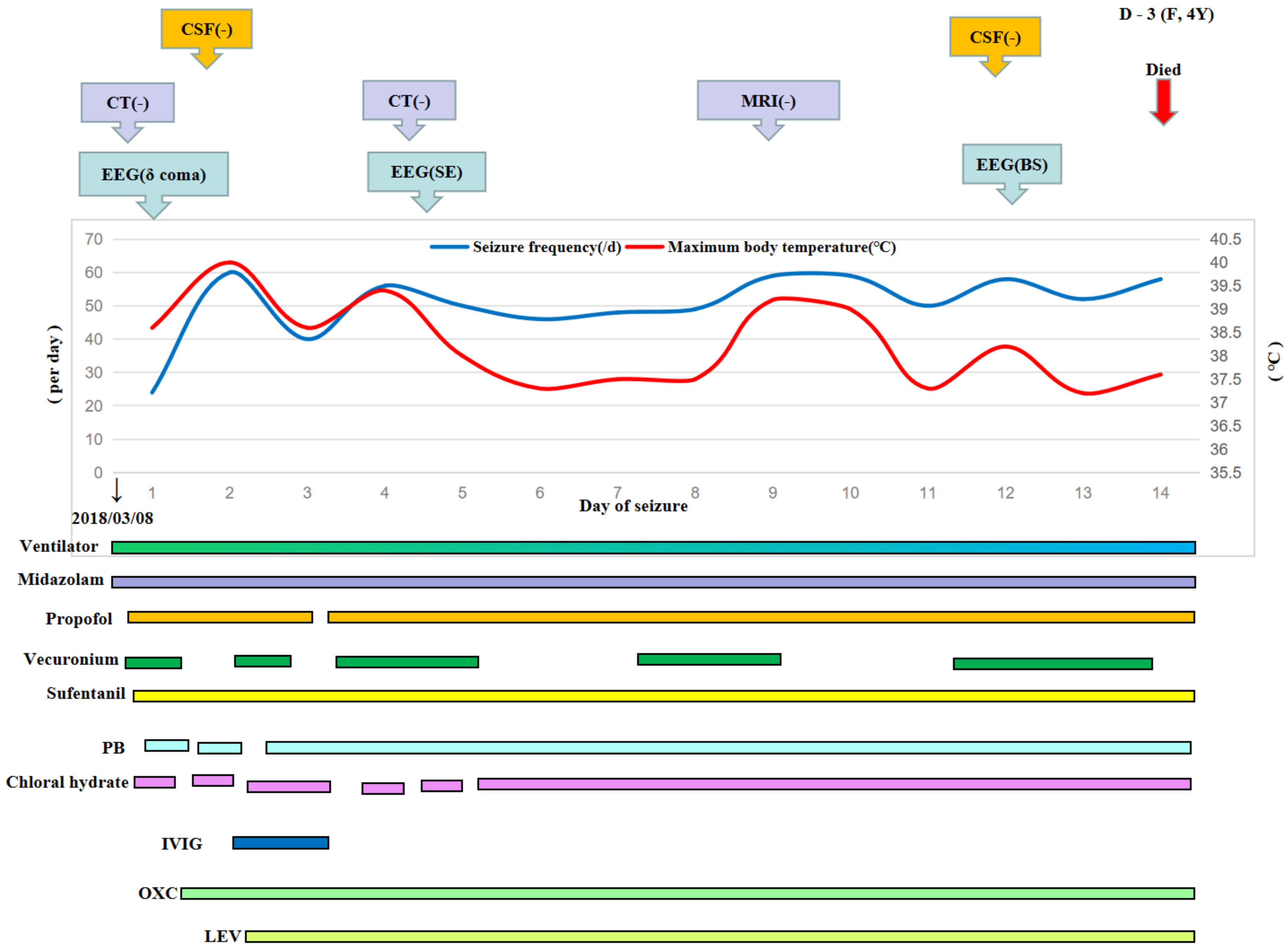

D - 3 (F, 4Y)
CSF(-)
CT(-)
EEG(δ coma)
CT(-)
EEG(SE)
MRI(-)
CSF(-)
EEG(BS)
Died
Seizure frequency(/d)
Maximum body temperature(℃)
(per day)
(℃)
70
60
50
40
30
20
10
0
40.5
40
39.5
39
38.5
38
37.5
37
36.5
36
35.5
1 2 3 4 5 6 7 8 9 10 11 12 13 14
Day of seizure
2018/03/08
Ventilator
Midazolam
Propofol
Vecuronium
Sufentanil
PB
Chloral hydrate
IVIG
OXC
LEV

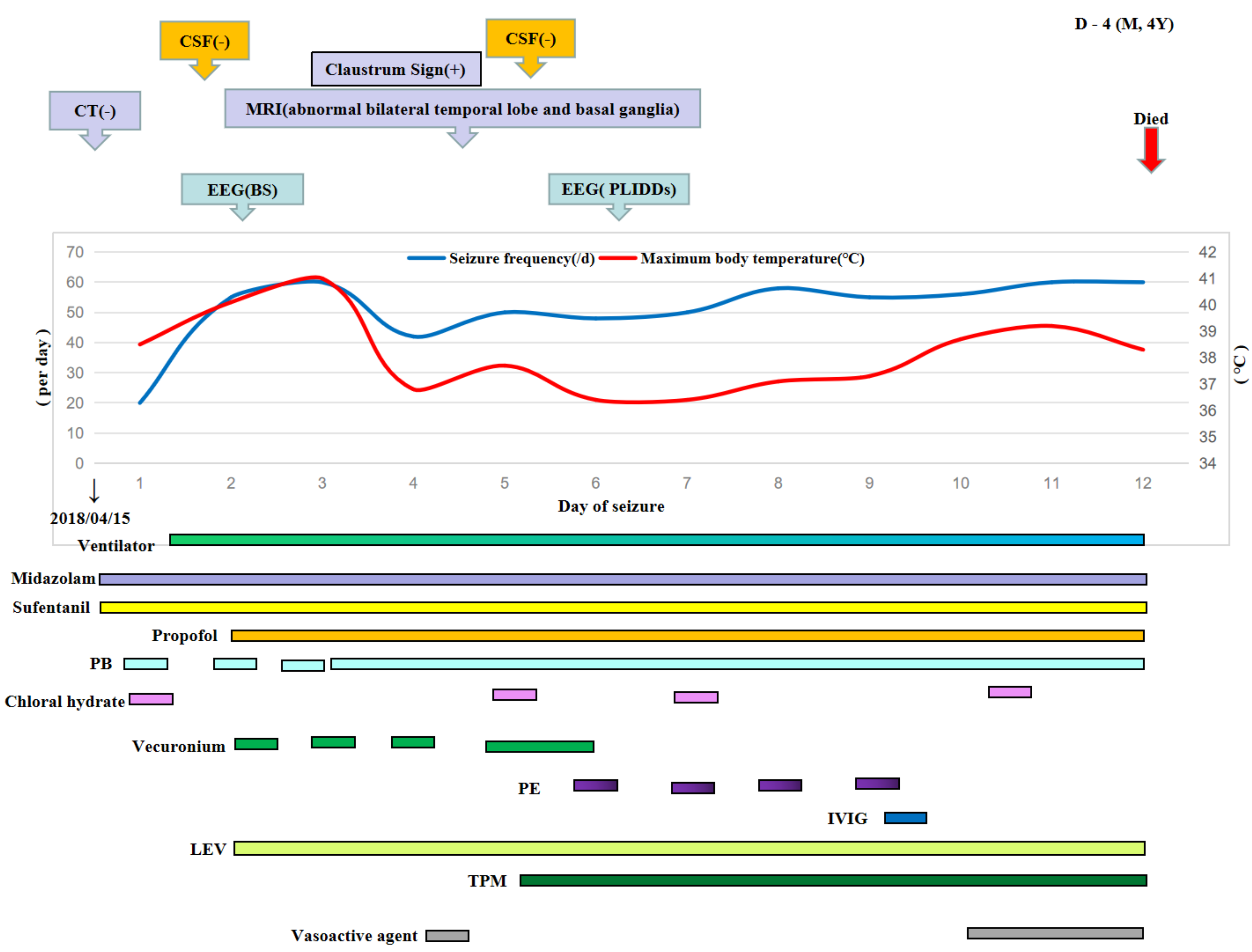

D - 4 (M, 4Y)
CSF(-)
CSF(-)
Claustrum Sign(+)
CT(-)
MRI(abnormal bilateral temporal lobe and basal ganglia)
Died
EEG(BS)
EEG(PLIDDs)
Seizure frequency(/d)
Maximum body temperature(℃)
(per day)
(℃)
Day of seizure
2018/04/15
Ventilator
Midazolam
Sufentanil
Propofol
PB
Chloral hydrate
Vecuronium
PE
IVIG
LEV
TPM
Vasoactive agent

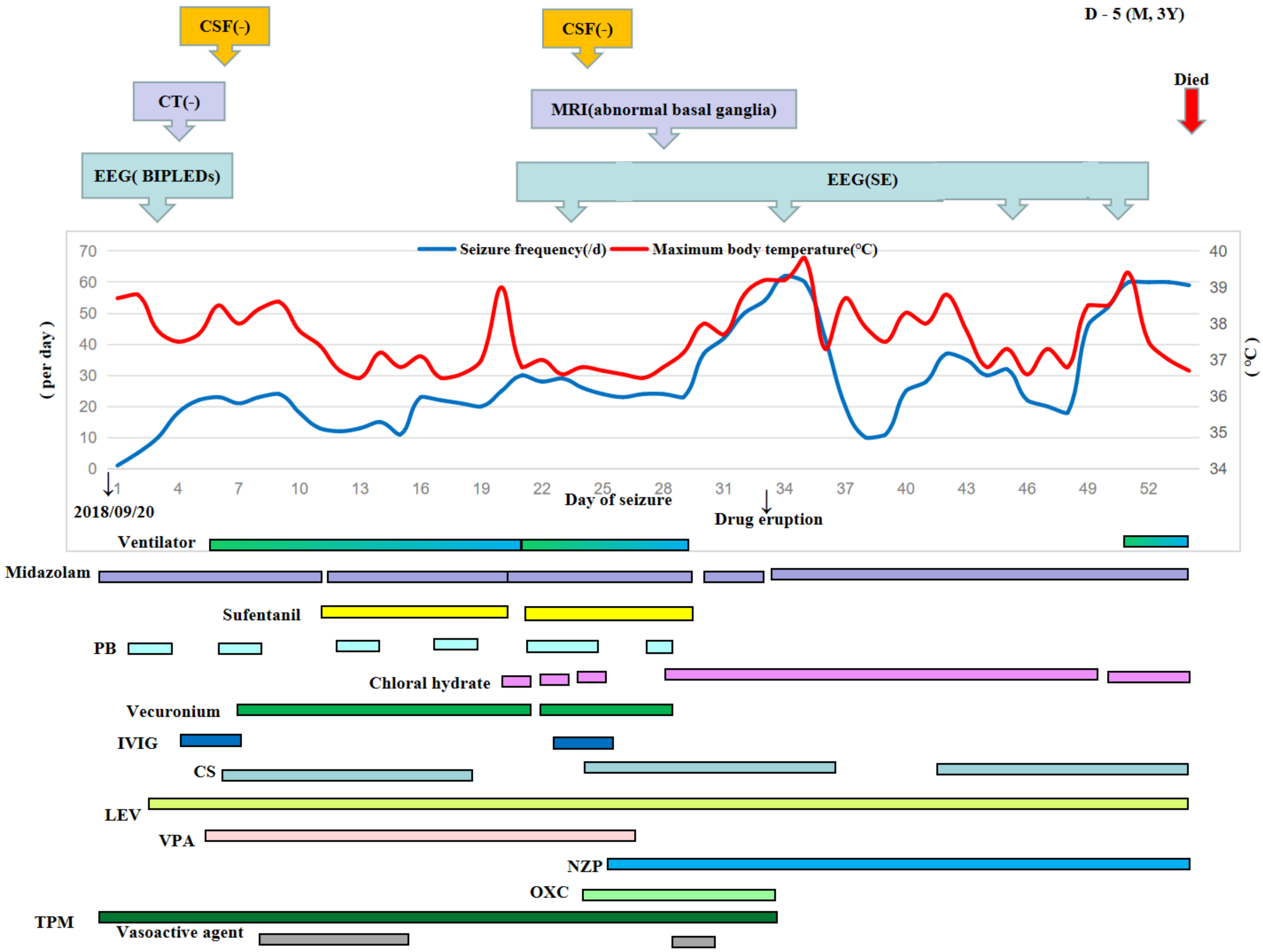

D - 5 (M, 3Y)
CSF(-)
CT(-)
EEG(BIPLEDs)
CSF(-)
MRI(abnormal basal ganglia)
EEG(SE)
Died
Seizure frequency(/d)
Maximum body temperature(°C)
(per day)
(°C)
Day of seizure
2018/09/20
Drug eruption
Ventilator
Midazolam
Sufentanil
PB
Chloral hydrate
Vecuronium
IVIG
CS
LEV
VPA
NZP
OXC
TPM
Vasoactive agent

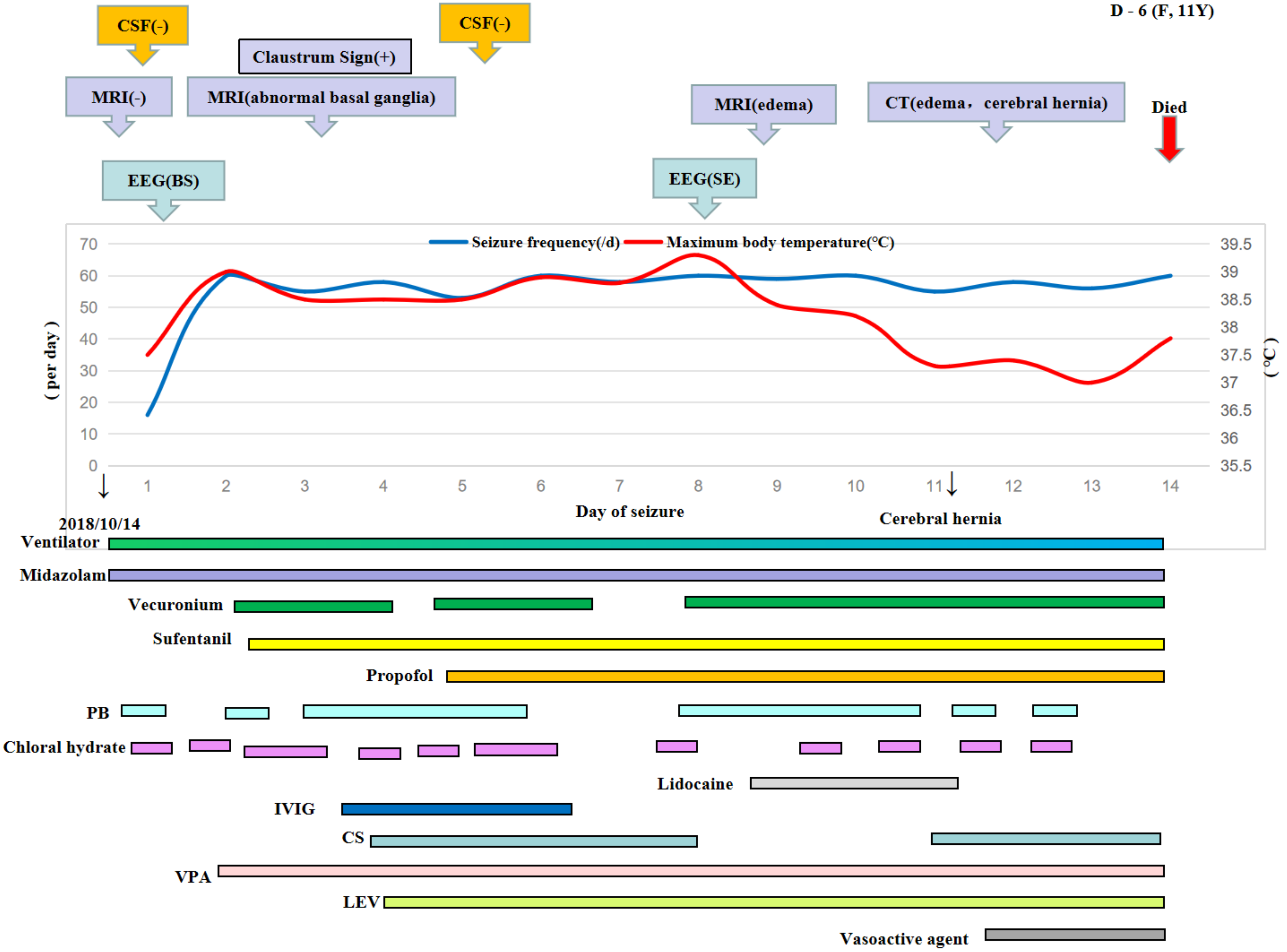
D - 6 (F, 11Y)
CSF(-)
Claustrum Sign(+)
CSF(-)
MRI(-)
MRI(abnormal basal ganglia)
MRI(edema)
CT(edema, cerebral hernia)
Died
EEG(BS)
EEG(SE)
Seizure frequency(/d)
Maximum body temperature(°C)
(per day)
(°C)
Day of seizure
2018/10/14
Cerebral hernia
Ventilator
Midazolam
Vecuronium
Sufentanil
Propofol
PB
Chloral hydrate
Lidocaine
IVIG
CS
VPA
LEV
Vasoactive agent

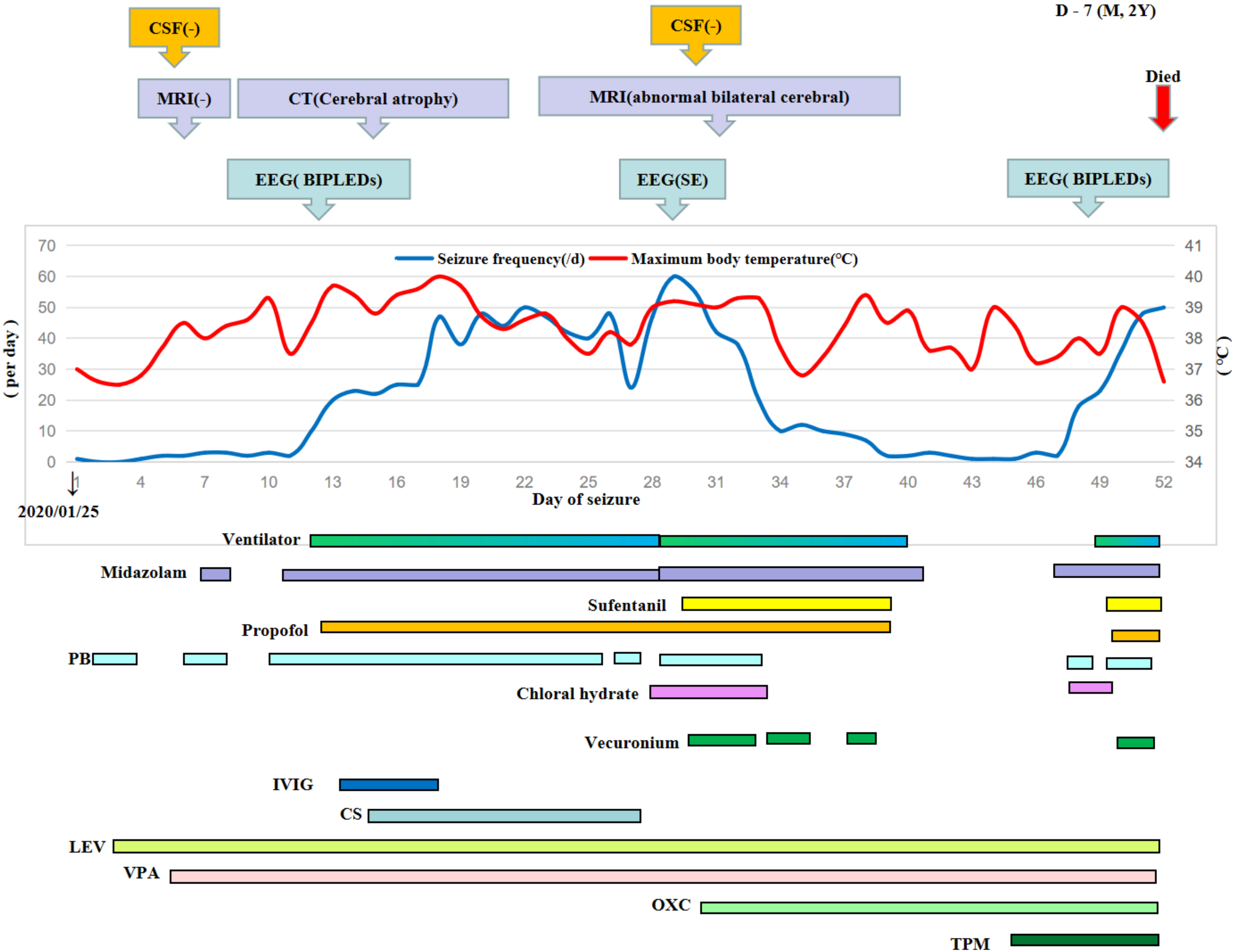

D - 7 (M, 2Y)
CSF(-)
MRI(-)
CT(Cerebral atrophy)
EEG(BIPLEDs)
CSF(-)
MRI(abnormal bilateral cerebral)
EEG(SE)
Died
EEG(BIPLEDs)
Seizure frequency(/d)
Maximum body temperature(℃)
(per day)
(℃)
Day of seizure
2020/01/25
Ventilator
Midazolam
Sufentanil
Propofol
PB
Chloral hydrate
Vecuronium
IVIG
CS
LEV
VPA
OXC
TPM

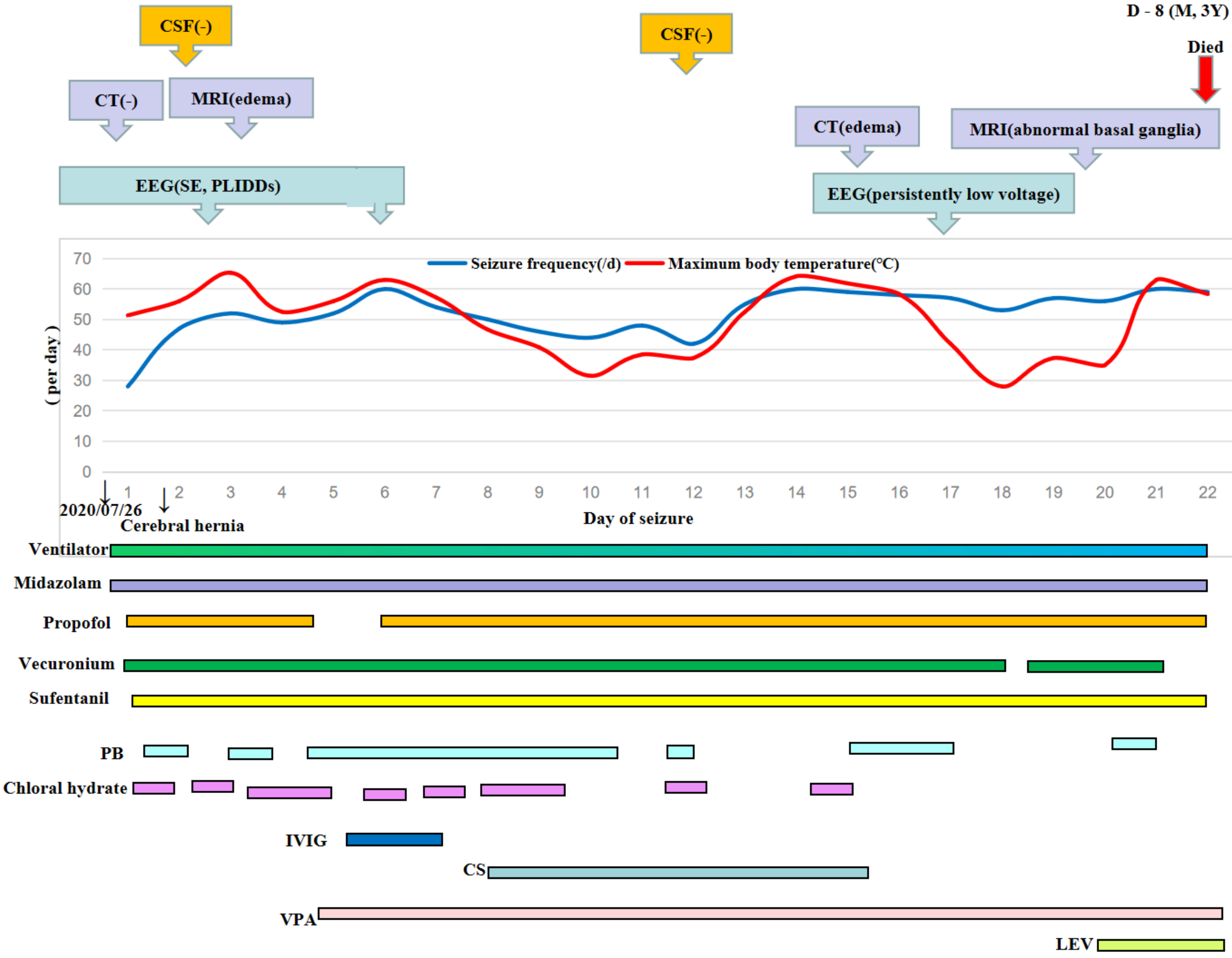

D - 8 (M, 3Y)
Died
CSF(-)
CSF(-)
CT(-)
MRI(edema)
CT(edema)
MRI(abnormal basal ganglia)
EEG(SE, PLIDDs)
EEG(persistently low voltage)
Seizure frequency(/d)
Maximum body temperature(°C)
(per day)
(°C)
70
60
50
40
30
20
10
0
40
39
38
37
36
35
34
1
2
3
4
5
6
7
8
9
10
11
12
13
14
15
16
17
18
19
20
21
22
2020/07/26
Cerebral hernia
Day of seizure
Ventilator
Midazolam
Propofol
Vecuronium
Sufentanil
PB
Chloral hydrate
IVIG
CS
VPA
LEV

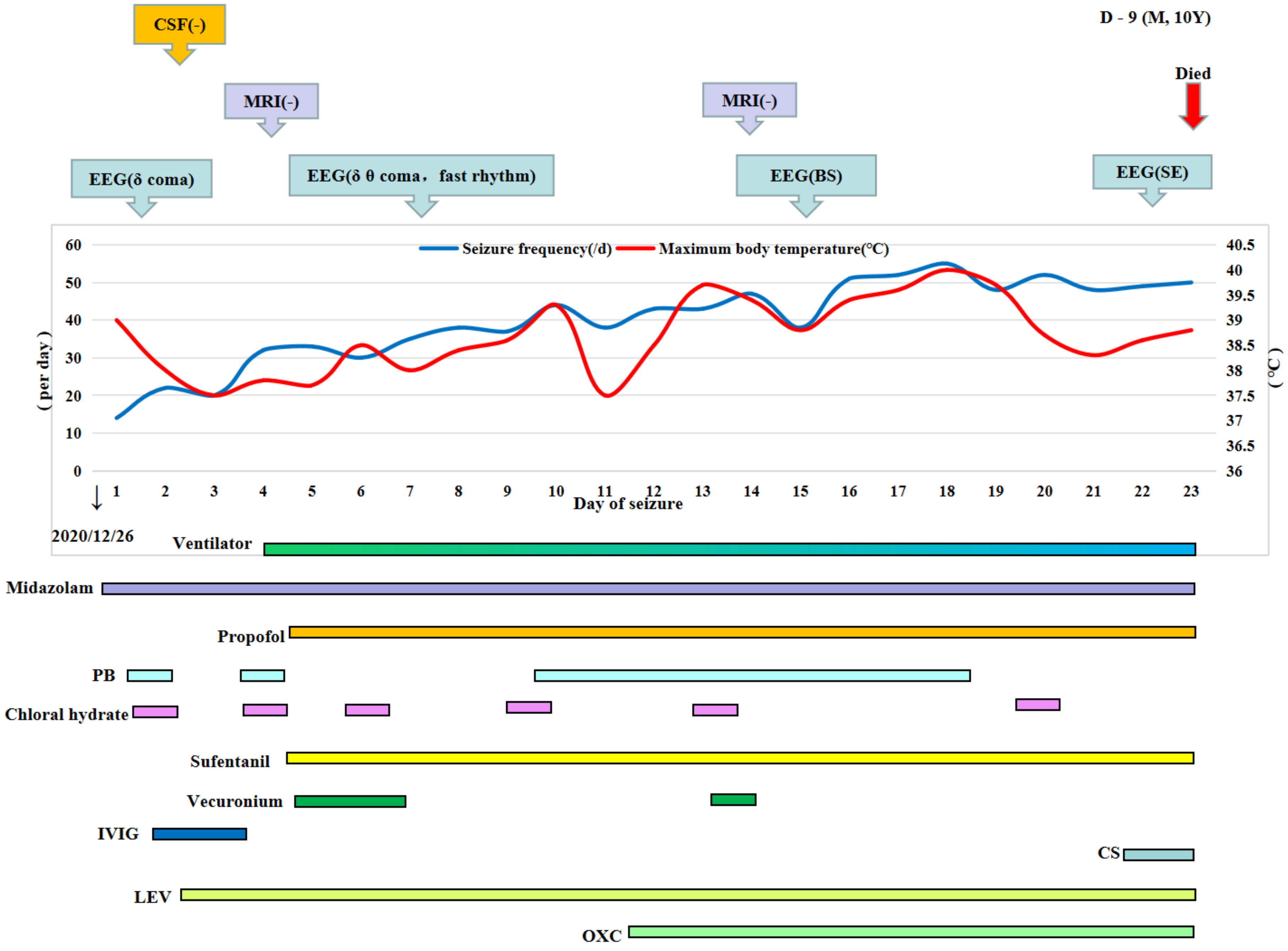

CSF(-)
D - 9 (M, 10Y)
Died
MRI(-)
MRI(-)
EEG(δ coma)
EEG(δ θ coma， fast rhythm)
EEG(BS)
EEG(SE)
Seizure frequency(/d)
Maximum body temperature(°C)
60
50
40
30
20
10
0
(per day)
40.5
40
39.5
39
38.5
38
37.5
37
36.5
36
(°C)
1 2 3 4 5 6 7 8 9 10 11 12 13 14 15 16 17 18 19 20 21 22 23
Day of seizure
2020/12/26
Ventilator
Midazolam
Propofol
PB
Chloral hydrate
Sufentanil
Vecuronium
IVIG
CS
LEV
OXC
