# Supplementary figures and images for: Clinical characteristics of 27 children with febrile infection‐related epilepsy syndrome in a single center
Source: Pediatr Discov. 2024 Jun 9;2(2):e84. doi: 10.1002/pdi3.84 (PMC12118283; doi:10.1002/pdi3.84)

**x: Maximum body temperature (°C)**

**y: Seizure frequency (/d)**

**— : Trendline**

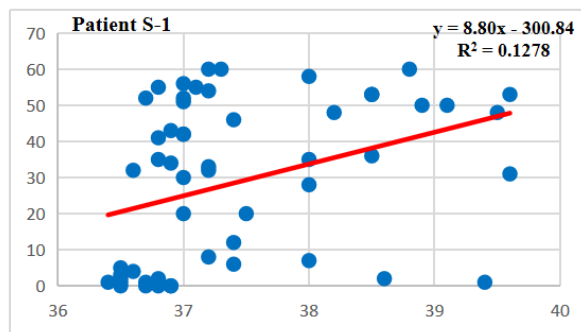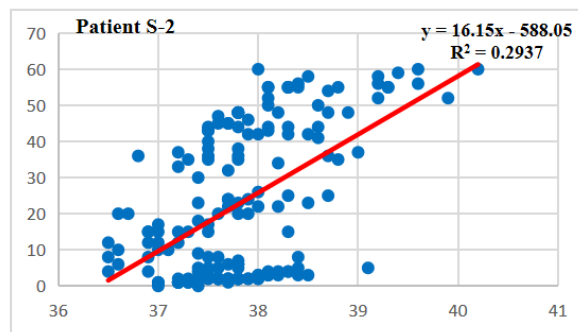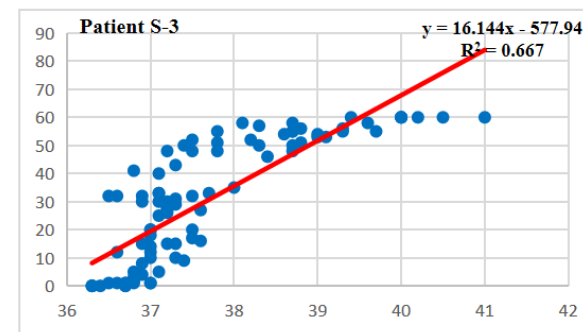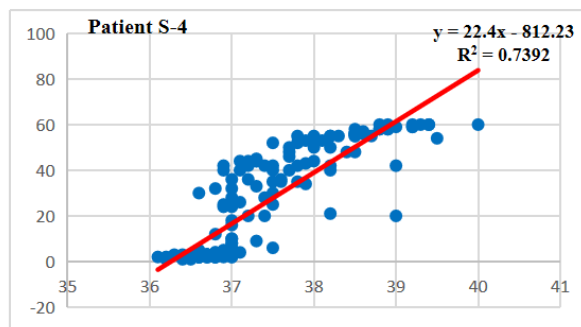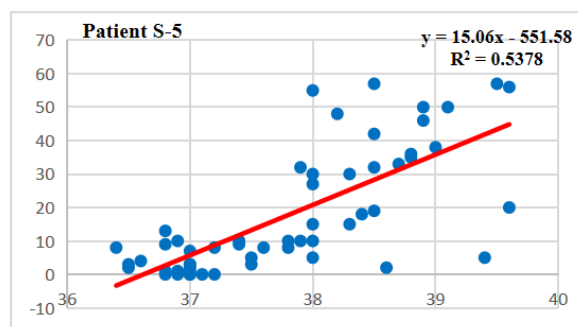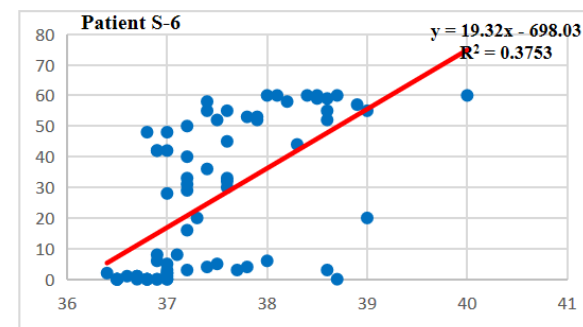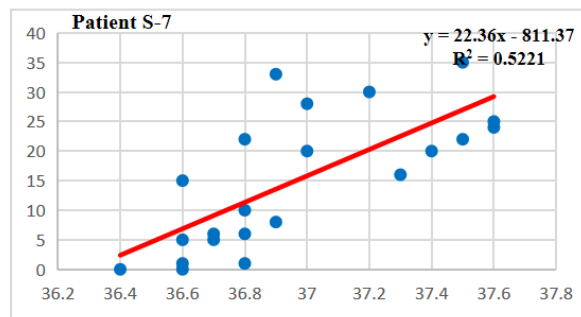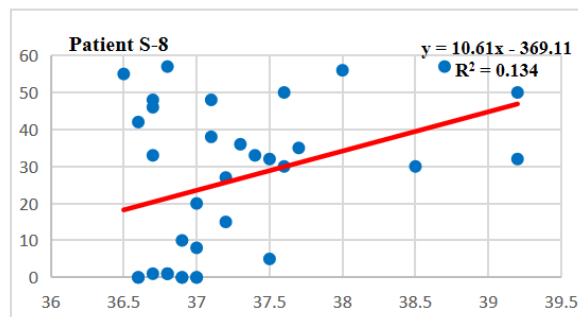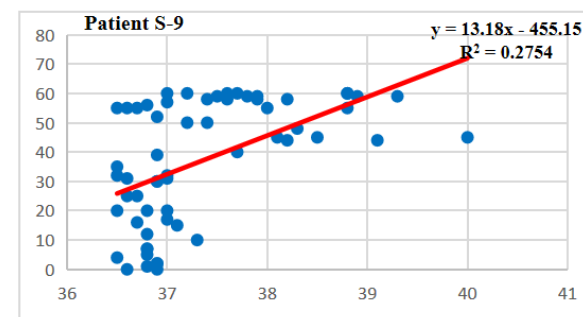

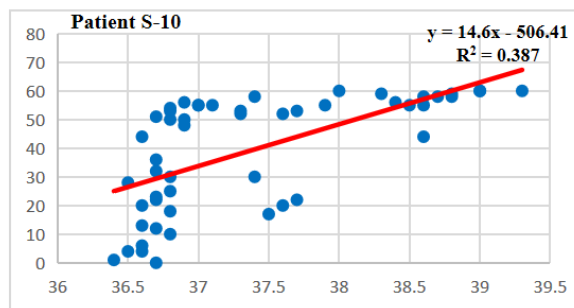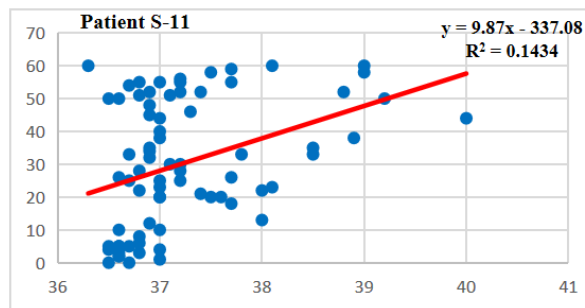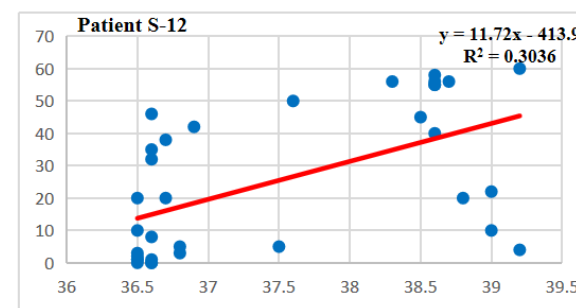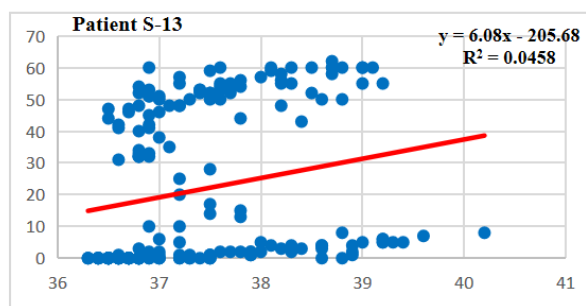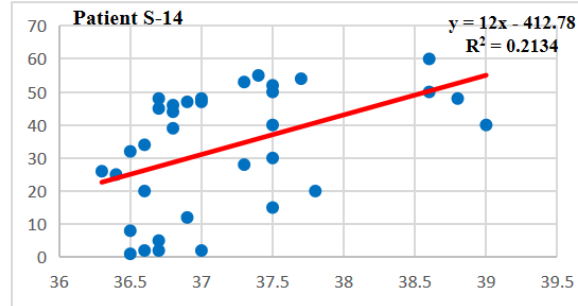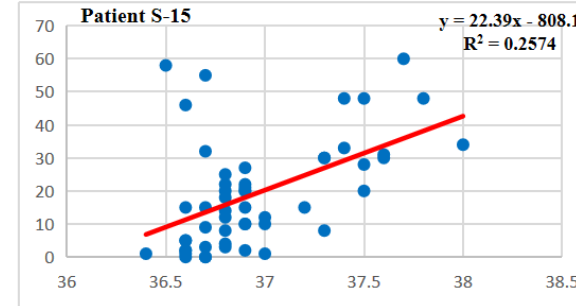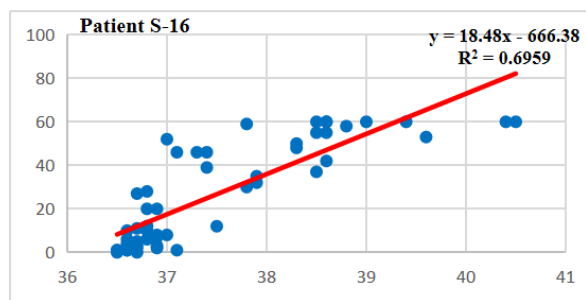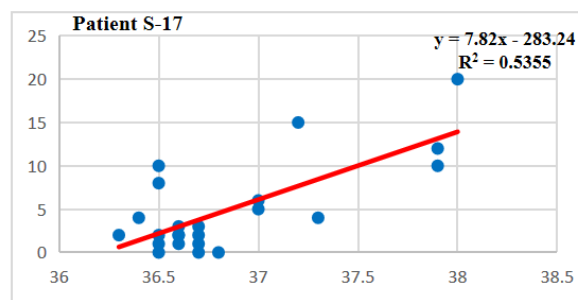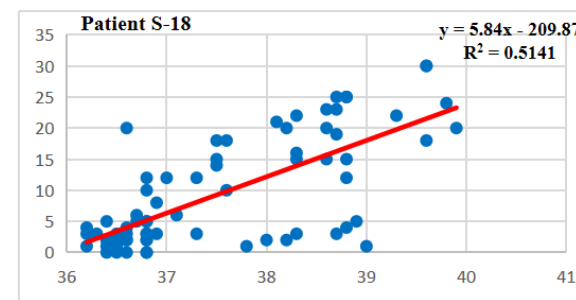

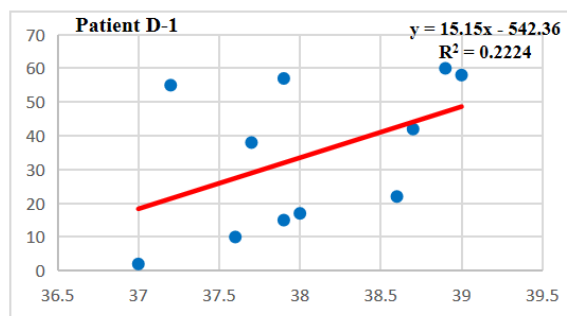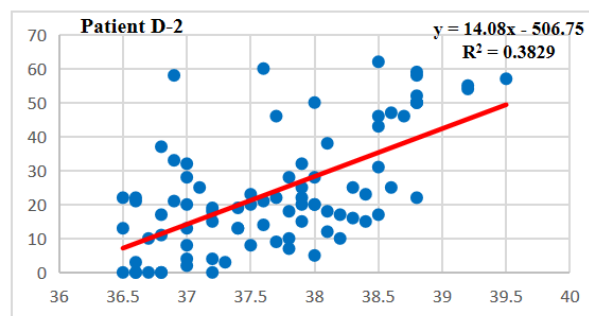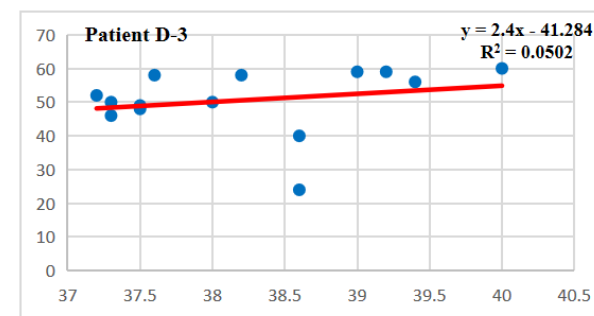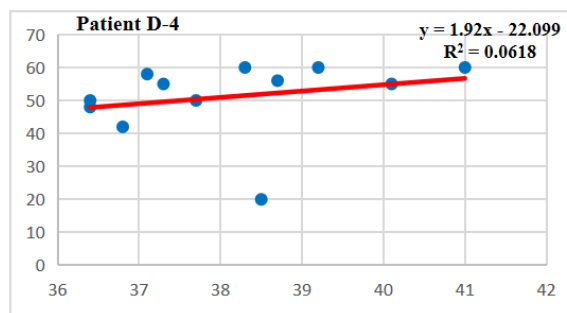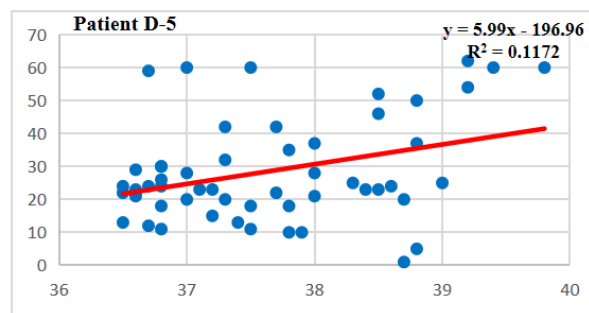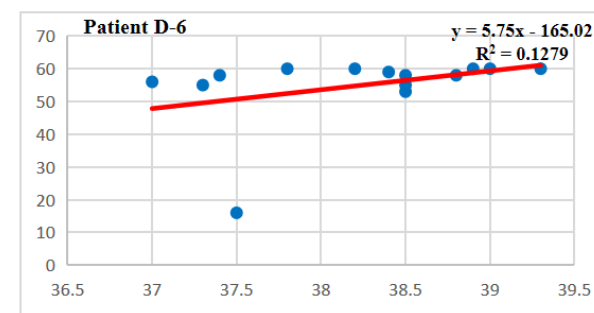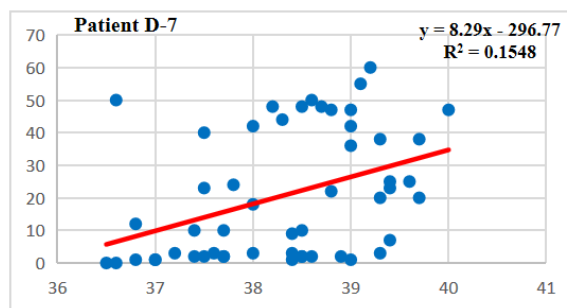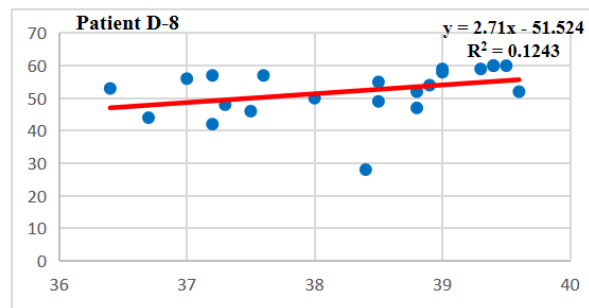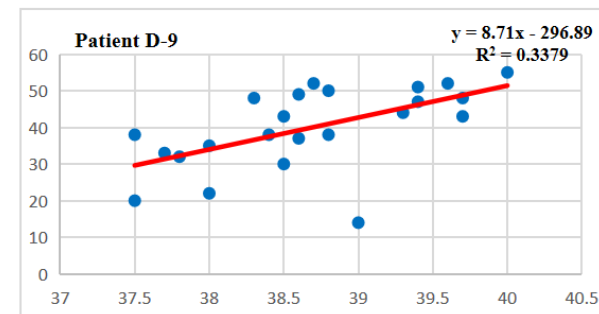

Supplement: Supplementary file 2 — Supplementary Material [file PDI3-2-e84-s001.pdf]
