# Supplementary material for: Clinical characteristics of 27 children with febrile infection‐related epilepsy syndrome in a single center
Source: Pediatr Discov. 2024 Jun 9;2(2):e84. doi: 10.1002/pdi3.84 (PMC12118283; doi:10.1002/pdi3.84)
Supplement: Supplementary file 3 — Table S1 [file PDI3-2-e84-s002.docx]

**Supplementary Table 1. Details regarding the characteristics of the two groups.**

|  |  |  |  | | FIRES group (n=7) | | | | | |  | |  | |  | |  | |  | | | non- FIRES group (n=7) | | | | | | | | | | | |  | | |  |  |  |
| --- | --- | --- | --- | --- | --- | --- | --- | --- | --- | --- | --- | --- | --- | --- | --- | --- | --- | --- | --- | --- | --- | --- | --- | --- | --- | --- | --- | --- | --- | --- | --- | --- | --- | --- | --- | --- | --- | --- | --- |
|  |  | Patient 1-1 | | Patient 1-2 | | Patient 1-3 | | Patient 1-4 | | Patient 1-5 | | Patient 1-6 | | Patient 1-7 | |  | | Patient 2-1 | | | Patient 2-2 | | | Patient 2-3 | | | Patient 2-4 | | | Patient 2-5 | | | Patient 2-6 | | | Patient 2-7 | | |  |
| Age (months) | | 98 | 79 | | 128 | | 102 | | 89 | | 109 | | 116 | |  | | 75 | | 168 | | | 147 | | | 122 | | | 77 | | | 70 | | | 109 | | | |  |  |
| Sex (male/female) | | M | M | | M | | M | | M | | M | | F | |  | | M | | M | | | M | | | F | | | M | | | M | | | M | | | |  |  |
| Duration of SE before KD(days) | | 90 | 66 | | 25 | | 34 | | 20 | | 14 | | 20 | |  | | 1820 | | 2520 | | | 1080 | | | 2520 | | | 1080 | | | 1080 | | | 1440 | | | |  |  |
| Anesthetic agents before KD | | Midazolam, Propofol | Midazolam, propofol | | Midazolam, propofol | | Midazolam, propofol | | Midazolam, propofol | | Midazolam, propofol | | Midazolam, propofol | |  | | Midazolam | | Midazolam | | | Midazolam | | | Midazolam | | | Midazolam | | | Midazolam | | | Midazolam | | | |  |  |
| Muscle relaxants before KD | | Vecuronium, fentanyl | Rocuronium, sufentanil | | Vecuronium, fentanyl | | Vecuronium, fentanyl | | Vecuronium | | Rocuronium | | Vecuronium | |  | | No | | No | | | No | | | No | | | No | | | No | | | No | | | |  |  |
| CS before KD | | Hydrocortisone→ methylprednisolone→ prednisone | Methylprednisolone→ prednisone | | Methylprednisolone→ prednisone | | Methylprednisolone→ prednisone | | Methylprednisolone→ prednisone | | Methylprednisolone→ prednisone | | Methylprednisolone→ prednisone | |  | | Methylprednisolone→ prednisone | | Methylprednisolone→ prednisone | | | Methylprednisolone→ prednisone | | | No | | | No | | | Methylprednisolone→ prednisone | | | Methylprednisolone→ prednisone | | | |  |  |
| IVIG before KD | | 4 g/kg | 4 g/kg | | 2 g/kg | | 4 g/kg | | 2 g/kg | | 2 g/kg | | 2 g/kg | |  | | 2 g/kg | | 2 g/kg | | | 2 g/kg | | | No | | | No | | | 2 g/kg | | | 2 g/kg | | | |  |  |
| PE before KD | | Yes | No | | Yes | | No | | No | | Yes | | No | |  | | No | | No | | | No | | | No | | | No | | | No | | | No | | | |  |  |
| KD ratio | | Classic 4:1 | Classic 4:1 | | Classic 4:1 | | Classic 2:1→LGIT | | Classic 3:1 | | Classic 4:1 | | Classic 2:1 | |  | | Classic 2:1 | | Classic 3:1 | | | Classic 2:1 | | | Classic 3:1 | | | Classic 3:1 | | | Classic 4:1 | | | Classic 2:1 | | | |  |  |
| Type of KD | | Intravenous → nasogastric tube → oral | Nasogastric tube → oral | | Nasogastric tube → oral | | Nasogastric tube → oral | | Nasogastric tube → oral | | Nasogastric tube → oral | | Nasogastric tube → oral | |  | | Oral | | Oral | | | Oral | | | Oral | | | Oral | | | Oral | | | Oral | | | |  |  |
| Supplementations | | Calcium 500 mg/d, vitamin D 600U/d | Calcium 800 mg/d, vitamin D 800U/d | | Calcium 500 mg/d, vitamin D 400U/d | | Calcium 500 mg/d, vitamin D 400U/d | | Calcium 500 mg/d, vitamin D 600U/d | | Calcium 500 mg/d, vitamin D 400U/d | | Calcium 500 mg/d, vitamin D 400U/d | |  | | Calcium 800 mg/d, vitamin D 400U/d | | Calcium 500 mg/d, vitamin D 800U/d | | | Calcium 500 mg/d, vitamin D 600U/d | | | Calcium 500 mg/d, vitamin D 800U/d | | | Calcium 500 mg/d, vitamin D 400U/d | | | Calcium 500 mg/d, vitamin D 400U/d | | | Calcium 500 mg/d, vitamin D 800U/d | | | |  |  |
| Duration of follow-up (months) | | 66 | 53 | | 24 | | 22 | | 19 | | 18 | | 15 | |  | | 66 | | 42 | | | 42 | | | 24 | | | 24 | | | 18 | | | 18 | | | |  |  |
| Duration of KD (months) | | 1 (stop) | 3 (stop) | | 9 (stop) | | 22  (continue) | | 19(continue) | | 11 (stop) | | 15 (continue) | |  | | 12(stop) | | 42(continue) | | | 18(stop) | | | 6(stop) | | | 9(stop) | | | 6(stop) | | | 18(continue) | | | |  |  |
| BOH (mmol/L) | | 2 | 1.2 | | 2 | | 3.6 | | 2 | | 1.4 | | 2.8 | |  | | 4.5 | | 4.1 | | | 4.9 | | | 3.5 | | | 4.7 | | | 3.2 | | | 3.8 | | | |  |  |
| Blood glucose  (mmol/L) | | 4.7 | 3.9 | | 4.2 | | 4.5 | | 3.8 | | 3.7 | | 3.6 | |  | | 3.8 | | 3.9 | | | 5.2 | | | 4.3 | | | 4.5 | | | 4.2 | | | 4.7 | | | |  |  |
| Duration off the anesthesia after KD(hours) | | 26 | 22 | | 27 | | 18 | | 19 | | 13 | | 10 | |  | | N/A | | N/A | | | N/A | | | N/A | | | N/A | | | N/A | | | N/A | | | |  |  |
| Duration of consciousness improvement after KD (days) | | 4 | 3 | | 3 | | 2 | | 4 | | 2 | | 2 | |  | | N/A | | N/A | | | N/A | | | N/A | | | N/A | | | N/A | | | N/A | | | |  |  |
| Duration off ventilator after KD (hours) | | 36 | 28 | | 17 | | 21 | | 24 | | N/A | | 15 | |  | | N/A | | N/A | | | N/A | | | N/A | | | N/A | | | N/A | | | N/A | | | |  |  |
| ASM before KD | | CZP/LEV/LTG/OXC/PHB | VPA/OXC/TPM/LEV | | OXC/LEV/VPA | | LEV/VPA | | OXC/TPM/VPA | | OXC/LEV | | VPA/LEV | |  | | VPA/LEV/TPM/NZP | | VPA/OXC/TPM | | | VPA/OXC/TPM/CZP | | | VPA/OXC/LEV/CZP | | | VPA/LEV/NZP | | | VPA/LEV/NZP/LTG | | | VPA/LEV/NZP/TPM | | | |  |  |
| ASM after KD at latest follow-up | | LEV/LTG/OXC | TPM | | TPM | | Drug withdrawal | | VPA | | Drug withdrawal | | VPA/LEV | |  | | LTG/CLB/ZNS | | ZNS/NZP/PB | | | VPA/TPM/CZP | | | TPM/LCM/CLB | | | TPM/CLB/LCM | | | TPM/ZNS/CLB | | | VPA/NZP/PHB | | | |  |  |
| Duration of improvement (≥50% seizure reduction) after KD (days) | | 14 | 30 | | 3 | | 4 | | 3 | | 2 | | 2 | |  | | 24 | | 28 | | | 5 | | | N/A | | | N/A | | | N/A | | | 7 | | | |  |  |
| Seizure burden at latest follow-up | | No seizure | No seizure | | No seizure | | No seizure | | No seizure | | No seizure | | Monthly seizures | |  | | Monthly seizures | | Weekly seizures | | | No seizure | | | Daily seizures | | | Daily seizures | | | Daily seizures | | | No seizure | | | |  |  |
| Cognition at latest follow-up | | Severe retardation | Moderate retardation | | Normal | | Normal | | Normal | | Normal | | Mild retardation | |  | | Mild retardation | | Severe retardation | | | Normal | | | Severe retardation | | | Severe retardation | | | Severe retardation | | | Normal | | | |  |  |
| mRS# score at latest follow-up | | 3 | 2 | | 0 | | 0 | | 0 | | 0 | | 1 | |  | | 2 | | 2 | | | 0 | | | 3 | | | 3 | | | 3 | | | 0 | | | |  |  |
|  | Background(Hz) | 2-3 | 2-4 | | 2-4 | | 2-4 | | 4-5 | | 3-4 | | 2-3 | |  | | 4-6 | | 4-5 | | | 5-7 | | | 5-6 | | | 4-6 | | | 3-4 | | | 3-5 | | | |  |  |
| EEG before KD | Inter - ictal | Frequent multifocal epileptiform discharges | Frequent multifocal epileptiform discharges | | Frequent multifocal epileptiform discharges | | Frequent multifocal epileptiform discharges | | Frequent multifocal epileptiform discharges | | Frequent multifocal epileptiform discharges | | Frequent multifocal epileptiform discharges | |  | | Frequent multifocal epileptiform discharges | | Epileptiform discharges | | | Epileptiform discharges | | | Frequent multifocal epileptiform discharges | | | Frequent multifocal epileptiform discharges | | | Frequent multifocal epileptiform discharges | | | Frequent multifocal epileptiform discharges | | | |  |  |
|  | Ictal | Electrographic and electroclinical  seizures | Electrographic and electroclinical  seizures | | Electroclinical seizures | | Electrographic and electroclinical  seizures | | Epileptiform electroclinical  seizures | | Electrographic and electroclinical  seizures | | Electrographic and electroclinical  seizures | |  | | Electroclinical seizures | | No | | | No | | | Electroclinical seizures | | | Electroclinical seizures | | | Electrographic seizures | | | Electroclinical seizures | | | |  |  |
|  | Seizure origin† | L/R/BF, BT, BO | L/R/BO, L/RT | | L/RP, L/RO, RC | | L/R/BO, L/RT,  LRolandic | | L/RP, L/RO, RC | | BF, BC, BT, BO | | LF, RO, L/R Rolandic | |  | | LO, LT | | No | | | No | | | LF, RO, RRolandic | | | LO, LT | | | BF, BC, BT, BO | | | L/RT, L/RF | | | |  |  |
|  |  |  |  | |  | |  | |  | |  | |  | |  | |  | |  | | |  | | |  | | |  | | |  | | |  | | | |  |  |
|  | Background(Hz) | 3-5 | 4-6 | | 8-9 | | 8-9 | | 8-10 | | 9-10 | | 5-7 | |  | | 4-6 | | 4-5 | | | 8-9 | | | 5-6 | | | 4-6 | | | 3-4 | | | 9-10 | | | |  |  |
| EEG at latest follow-up | inter - ictal | Epileptiform discharges | Normal | | Normal | | Normal | | Normal | | Normal | | Epileptiform discharges | |  | | Epileptiform discharges | | | Epileptiform discharges | | | Normal | | | Frequent multifocal epileptiform discharges | | | Frequent multifocal epileptiform discharges | | | Frequent multifocal epileptiform discharges | | | Normal | | | | |
|  | ictal | No | No | | No | | No | | No | | No | | No | |  | | No | | Electroclinical seizures | | | No | | | Electrographic and electroclinical seizures | | | Electrographic  seizures | | | Electrographic and electroclinical seizures | | | No | | | |  |  |
|  | Seizure origin† | No | No | | No | | No | | No | | No | | No | |  | | No | | B Rolandic | | | No | | | LF, RO, Rrolandic | | | LO, BT | | | BF, BC, BT, BO | | | No | | | |  |  |
| Adverse events | | Hyperlipidemia | No | | Vomit | | No | | No | | No | | No | |  | | No | | No | | | No | | | No | | | No | | | No | | | No | | | |  |  |

SE status epilepticus; KD ketogenic diet; CS, corticosteroids; IVIG, intravenous immunoglobulins; PE, plasma exchange; ASM, anti-seizure medication; CZP, clonazepam; LEV, levetiracetam; LTG, lamotrigine; OXC, oxcarbazepine; PHB, phenobarbital; VPA, valproic acid; TPM, topiramate; NZP, nitrazepam; ZNS, zonisamide; LCM, lacosamide; phenobarbital. BOH blood serum β-hydroxybutyrate; EEG, electroencephalogram

^#^ mRS: modified Rankin Scale

^†^ Seizure origin: L, left; R, right; B, bilateral; F, frontal; T, temporal; C, central; P, parietal; O, occipital;
